# Long-read-based Genome Assembly of *Drosophila gunungcola* Reveals Fewer Chemosensory Genes in Flower-breeding Species

**DOI:** 10.1093/gbe/evad048

**Published:** 2023-03-17

**Authors:** Ateesha Negi, Ben-Yang Liao, Shu-Dan Yeh

**Affiliations:** Department of Life Sciences, National Central University, Taoyuan City, Taiwan, Republic of China; Institute of Population Health Sciences, National Health Research Institutes, Zhunan, Miaoli County, Taiwan, Republic of China; Department of Life Sciences, National Central University, Taoyuan City, Taiwan, Republic of China

**Keywords:** *Drosophila gunungcola*, gene annotation, PacBio sequencing, chemosensory genes

## Abstract

*Drosophila gunungcola* exhibits reproductive activities on the fresh flowers of several plant species and is an emerging model to study the co-option of morphological and behavioral traits in male courtship display. Here, we report a near-chromosome-level genome assembly that was constructed based on long-read PacBio sequencing data (with ∼66× coverage) and annotated with the assistant from RNA-seq transcriptome data of whole organisms at various developmental stages. A nuclear genome of 189 Mb with 13,950 protein-coding genes and a mitogenome of 17.5 kb were acquired. Few interchromosomal rearrangements were found in the comparisons of synteny with *Drosophila elegans*, its sister species, and *Drosophila melanogaster*, suggesting that the gene compositions on each Muller element are evolutionarily conserved. Loss events of several OR and IR genes in *D. gunungcola* and *D. elegans* were revealed when orthologous genomic regions were compared across species in the *D. melanogaster* species group. This high-quality reference genome will facilitate further comparative studies on traits related to the evolution of sexual behavior and diet specialization.

**Significance**

A high-quality and well-annotated genome in *Drosophila gunungcola* is vital to comparative and population genomics studies on the evolution of morphology, behavior, diet, niche shift, and karyotypes that are unique in the *Drosophila elegans* species group. We generated a near-chromosomal-level and well-annotated reference genome for studying molecular basis underlying fruit fly niche adaptation. This assembled genome allowed us to accurately identify boundaries of gene loss events, including those of chemosensory receptors, and suggest the potential role of the loss of a specific OR gene that has played in the ecological specialization for flower-breeding behaviors. Our findings demonstrated that available genome resources of the second species in the *elegans* species group increased the power to detect genetic changes in the evolution of novel traits.

## Introduction

**Fig. 1.**
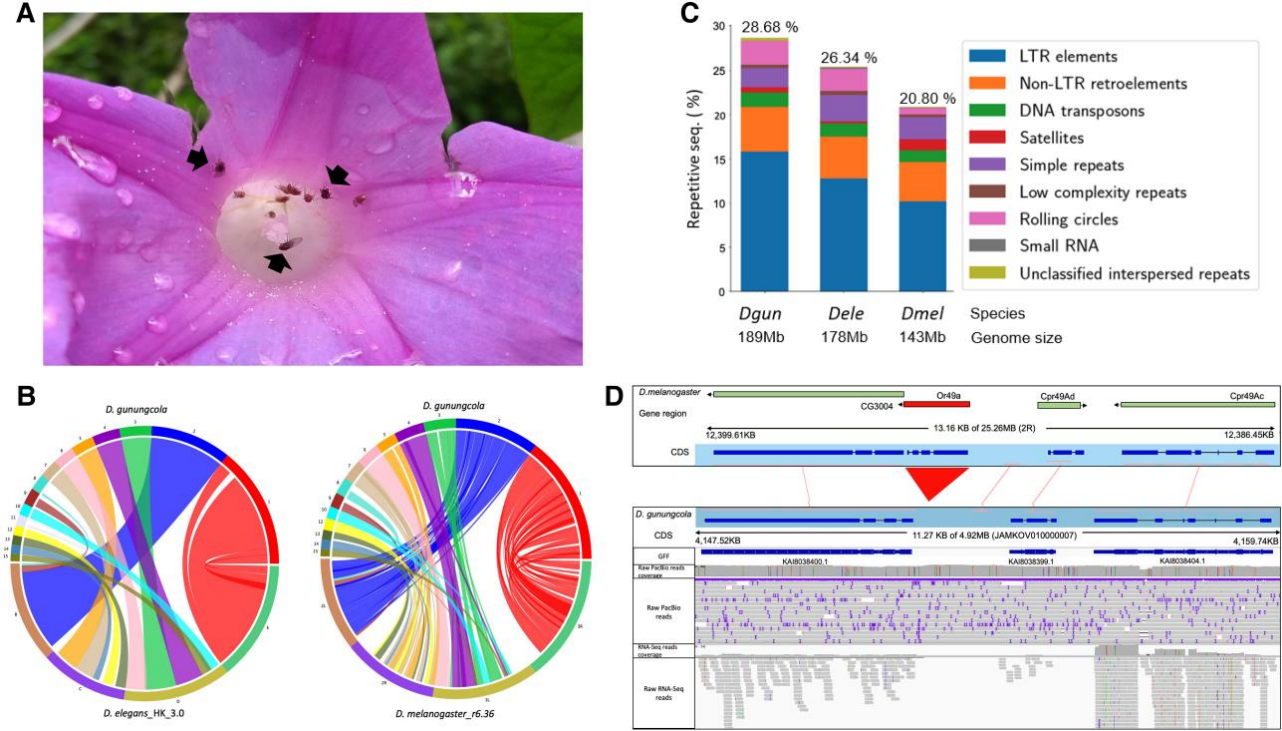
The genome information of *D. gunungcola*. (*A*) *Drosophila gunungcola*, indicated by arrows, coexists with *D. elegans*, unmarked fly individuals, on an *I. indica* flower in the sympatric area. The photo was taken in Sumatra, in 2018 by S.-D.Y. (*B*) Synteny of the 15 longest contigs assembled in this study with *D. elegans* and *D. melanogaster* chromosomes. The comparisons on the left are the contigs of *D. gunungcola* (on the top) and 4 major autosomes of *D. elegans* (GCA_018152815.1). The comparisons on the right are the contigs of *D. gunungcola* (on the top) and 4 major autosome arms of *D. melanogaster* (r6.36) (on the bottom). Each color represents a contig of *D. gunungcola* automatically assigned by SyMAP. (*C*) The proportions of various types of repetitive sequences in *D. gunungcola* (assembled in this study), *D. elegans* (GenBank assembly accession: GCA_018152505.1), and *D. melanogaster* (r6.36; FlyBase) genomes. (*D*) *OR49a* loss in *D. gunungcola*. *OR49a* gene region, indicated in the red triangle, along with adjacent genes in *D. melanogaster* 13 kb in the upper panel schematically align to *D. gunungcola* 11 kb in the lower panel. The protein sequences of *CG30048*, *Cpr49Ad*, and *Cpr49Ac* are 51, 90, and 87% identical to the protein sequences encoded in KAI8038400.1, KAI8038399.1, and KAI8038404.1, respectively. Raw PacBio reads extending two adjacent genes support the correct assembly of this region and raw RNA-seq reads mapping to this region support the gene annotation. The upper and lower panels of graphs were generated in SyMAP and IGV, respectively.

*Drosophila gunungcola* is one of the five described species in the *elegans* species subgroup under the *D. melanogaster* species group of *Sophophora* subgenus in *Drosophila* (Sultana et al. 1999; Ishikawa et al. 2022). This species and its sibling species, *D. elegans*, breed on tubular flowers of species in *Ipomoea* (morning glories), *Brugmansia* (Angel's trumpet), and *Hibiscus* (Hirai and Kimura 1997; Sultana et al. 1999; Ishikawa et al. 2022). Male and female adults of these two species visit flowers for mating and oviposition (fig. 1*A*), contrasting to most species of the *D. melanogaster* species group that perform reproductive activities on the fruits or fermenting substrates. The unique characteristics of the *elegans* species subgroup go beyond its diet and habitat as this clade is nested within the *D. melanogaster* species group. Unlike the stereotypic karyotype of four chromosome pairs (2*n* = 8) in the *D. melanogaster* species group, the *elegans* species subgroup consists of five telocentric chromosome pairs and a subtelocentric sex chromosome pair (2*n* = 12) (Deng et al. 2007; Yeh and True 2014; Ishikawa et al. 2022). Each chromosome corresponding to a Muller element and the position of centromeres suggests the maintenance of the ancestral karyotype after divergence of the *elegans* species subgroup (Ferreri et al. 2005; Deng et al. 2007). Furthermore, the contrasting differences of multiple traits in a closely related and hybridizable species pair, *D. gunungcola* and *D. elegans*, within the *elegans* species subgroup become important in studying the rapid evolution of male sexual characteristics related to mating success, wing spots, and wing display, as well as body melanization related to ecological adaptation (Kopp and True 2002; Yeh et al. 2006; Yeh and True 2014; Massey et al. 2020; Ishikawa et al. 2022). A well-annotated genome of *D. gunungcola* becomes vital for further dissecting the genetic and molecular mechanisms in these studies.

Despite the growing popularity of including *D. gunungcola* in various research, this species lacks a highly contiguous and annotated reference genome. To date, the only available genome in *D. gunungcola* is short-read-based, composed of 5,250 scaffolds, highly fragmented and, more seriously, unannotated (Massey et al. 2020). Thus, we intended to assemble a chromosome-level genome of *D. gunungcola* using long-read sequencing and annotate this genome with RNA-seq data. Our assembly offers us an opportunity to study the influences of structural DNA changes (e.g., deletions, duplications, and rearrangements) in organismal adaptation. For instance, focusing on two groups of chemosensory receptor genes, odorant receptors (ORs), and ionotropic receptors (IRs), we identified species-specific gene deletion events that might have played an important role for flower-breeding species (i.e., *D. gunungcola* and *D. elegans*) in occupying divergent ecological niches.

## Results and Discussion

### Near-Chromosomal Assembled Genome of *D. gunungcola*

To obtain a contiguous genome assembly, 10.76 Gb long-read sequencing data with N50 of 9,020 bp were acquired using genomic DNAs extracted from *D. gunungcola* male thorax (see Materials and Methods). No xenobiotic sequences, that is, from *Drosophila* gut bacteria and yeasts, were detected in the initial assemblies, suggesting that the genomic DNA extracted from thoracic tissue was free of such contaminates. We obtained a 189.84-Mb genome, including nuclear and mitochondrial sequences, in 351 contigs with N50 of 3,022,255 bases and the longest contig of 31,037,916 bases after three rounds of merging processes and haplotype removal (table 1, supplementary Materials and Methods, Supplementary Material online). Compared to the 178.44 Mb of the *D. elegans* female genome, this assembled *D. gunungcola* genome is 11.4 Mb larger. This size difference between two assemblies is close to ∼14.6 Mb of the *D. melanogaster* Y chromosome (Chang and Larracuente 2019), suggesting that the additional sequence is mainly from Y chromosome in our assembled *D. gunungcola* genome. The size of the assembled genome is generally in agreement with 0.19–0.2 pg (190–200 Mb) of the *D. elegans* genome in a flow cytometry study (Gregory and Johnston 2008; de Lima and Ruiz-Ruano 2022).

**Table 1:** Summary of *D. gunungcola* Genome Assembly and Annotation Statistics in Comparison to the Previous WGS-Based Assembly Available in NCBI Database (GenBank Assembly Accession: GCA_011057485.1) and Its Sister Species *D. elegans* (GenBank Assembly Accession: GCA_018152505.1)

Description	*D. gunungcola* (assembled in this study)	*D. gunungcola* (D_gunungcola_WGS_1.0)	*D. elegans* (ASM1815250v1)
Sex	Male	Female	Female
Sequencing technology	PacBio Sequel	Illumina HiSeq	Oxford Nanopore MinION and Illumina GAIIX
Genome coverage	66.2X	20.0X	108.2X
Assembly size (Mb)	189.84	168.09	178.44
Number of contigs/scaffolds	351 (primary contigs)	5,421 (scaffolds)	720(contigs)
Contig belonging to mitogenome	1(17,498 bp, complete)	N/A	N/A
Gap per (N's) 100 kb	0	3,556.17	0
N50 (bp)	3,022,255	1,718,255	21,933,072
Longest contig/scaffold (Mb)	31.03	7.42	26.85
GC content	39.78%	40.33%	39.55%
Assembly completeness (BUSCOs = 3285)	98.8% (3244)	98.3% (3230)	99.3% (3262)
Complete and single copy	98.5% (3236)	98.1% (3222)	99.1% (3256)
Complete and duplicated	0.2% (8)	0.2% (8)	0.2% (6)
Fragmented	0.4% (12)	0.5% (17)	0.1% (3)
Missing	0.9% (29)	1.2% (38)	0.6% (20)
Annotated protein completeness (BUSCOs = 3285)	87.8% (2884)	N/A	89.3% (2932)
Complete and single copy	87.3% (2869)	N/A	79.2% (2601)
Complete and duplicated	0.5% (15)	N/A	10.1% (331)
Fragmented	4.8% (157)	N/A	3.7% (120)
Missing	7.4% (244)	N/A	7.1% (233)
Contigs belonging to autosomes	28 (105 Mb)	N/A	N/A
Contigs belonging to X/Y chromosome	66 (20 Mb)	N/A	N/A
Unclassified contigs	256 (64 Mb)	N/A	N/A
Nuclear protein-coding genes	13,950	N/A	14,058
Nuclear tRNA genes	315	N/A	288
Mitochondria protein-coding genes	12	N/A	N/A
Repeat annotation (%)	28.68%	N/A	26.34%

A total of 13,950 protein-coding genes were annotated in this assembled genome by using RNA-seq data from five whole-body samples and protein sequences of eight *Drosophila* species (see Materials and Methods). Out of 3,285 Benchmarking Universal Single-Copy Orthologs, BUSCOs, only 29 and 12 BUSCOs were missing and fragmented in this assembled genome, respectively. The proportion of BUSCOs that present in full length in our assembled genome is 98.8%, which is comparable to *D. melanogaster* r6.36 (99.4%, 3265/3285) and *D. elegans* ASM1815250v1 (99.3%) (table 1). When the first 15 longest contigs, accounting for 101 Mb in total, were aligned to *D. elegans*_HK_3.0 and *D. melanogaster* r6.36, none of them match more than one chromosome (fig. 1*B*). The one-to-one match between our contigs and *D. melanogaster* genome suggests the least probable misassembly since the gene composition of a Muller element is usually well conserved in *Drosophila* (Ranz et al. 2003). Compared to the gene composition in conserved dipteran orthologs and the *D. elegans* genome, the longest contig with 31.03 Mb covers the full length of Muller element E (supplementary fig. S1, Supplementary Material). The rest of the autosomes is composed of as few as 27 contigs, suggesting that this reference-quality genome is at a near-chromosomal level. Nonetheless, the highly fragmented X chromosome spanning 66 contigs may result from the combination of repetitive sequences and high sequence similarity to the Y chromosome (supplementary fig. S2, Supplementary Material).

### Long-Read Contiguous Assembly Resolves the Previous Misassembly

In comparison to the previous Illumina-based genome assembly using the same strain as used in this study (Massey et al. 2020; D_gunungcola_WGS_1.0; GeneBank: GCA_011057485.1; sequencing depth = 20.0X), the higher sequencing depth and long reads of our PacBio sequencing data allowed a better genome assembly as indicated by less N's gaps, fewer *N* bases, and smoother distribution of GC content (table 1, supplementary fig. S3, Supplementary Material). Our assembly recovered 27 complete single-copy dipteran orthologs that were missing in the previous assembly, although 18 BUSCOs found in the previous Illumina-based assembly are missing in our assembly (supplementary table S1, Supplementary Material). Inversions and translocations detected in the syntenic comparisons of the previous and our assemblies are mostly due to the manual concatenation and lack of read coverage near the breaking points in the previous assembly (supplementary figs. S4 and S5, Supplementary Material). Overall, a well-annotated and contiguous genome at the near-chromosomal level was presented in this study.

### More Repetitive Sequence in *D. gunungcola* and *D. elegans*

Approximately 54.44 Mb (28.68%) in the *D. gunungcola* genome was found to be repetitive sequences, which is comparable to the repeatome in *D. elegans* (45.23 Mb, 26.34%) but considerably larger than that in *D. melanogaster* (29.89 Mb, 20.80%) (fig. 1*C*). Most of the repetitive sequences (72.68%) were retrotransposons, especially the LTR elements (supplementary table S2, supplementary file, Supplementary Material online). A positive correlation between the genome size and TE content has been reported in the species of *Sophophora* subgenus although the genome size differences are influenced by both repetitive and nonrepetitive portions of the genome (Kim et al. 2021). Therefore, the repetitive sequences may partially contribute to the underlying genome size differences between the *elegans* species subgroup and *D. melanogaster*.

### Gene Loss of the Olfactory Receptor Gene Families in *D. gunungcola*

The availability of well-assembled and annotated genomes provides an opportunity to study the genetic basis associated with behavioral diversification in evolutionary ecology within the *D. melanogaster* species group. Two main types of olfactory receptor genes, encoding ORs and IRs, are of great interest because of their possible roles in the recognition of breeding substrates (Clyne et al. 1999; Benton et al. 2009). ORs detect a vast array of food-related chemicals and are highly adapted to recognize airborne odorants like alcohols and esters (Hallem et al. 2004; Couto et al. 2005; Brand et al. 2018). IRs are anciently originated chemosensory receptors and are highly adapted to recognize acids and amines (Croset et al. 2010; Silbering et al. 2011; Min et al. 2013; Missbach et al. 2014). We identified fewer genes belonging to the two gene families in *D. gunungcola* than in *D. elegans* (55 vs. 56 for ORs, 55 vs. 60 for IRs) as well as compared to seven other species within the *D. melanogaster* species group (supplementary tables S3 and S4, Supplementary Material). We confirmed the OR and IR gene loss by examining the synteny of adjacent orthologs (supplementary fig. S6, Supplementary Material). The complete lack of *Ir11a* solely in *D. gunungcola* and lack of *Or49a* and *Ir94a-Ir94b-Ir94c* both in *D. gunungcola* and *D. elegans* are supported by the raw PacBio reads extending in the regions. More pseudogenes derived from IRs and ORs were found in the current genome of *D. gunungcola*, in comparison with those found in the genome of *D. elegans*, suggesting that the fewer IR and OR genes found in the *D. gunungcola* genome are not an artifact which resulted from potential genome incompleteness (four vs. one). Fewer ORs and IRs have also been found in the genome of *Drosophila sechellia*, which is known to specialize in feeding on *Miranda* fruit, indicating a trend of gene loss during ecological specialization.

*Or49a*, encoding receptor associating with 1-hexanol detection and parasitoid avoidance behavior, is exclusively absent in the flower-breeding sibling species, but present in the rest of the recently studied *Sophophora* species (Fishilevich and Vosshall 2005; Ramasamy et al. 2016). Our detailed investigation of the genomic region adjacent to *Or49a* and long-read data also supported the loss of *Or49a* (fig. 1*D*), indicating that the recent loss of *Or49a* in the *elegans* species subgroup might be associated with its specialization of flower breeding. However, the loss of *Or49a* in these two species needs to be further confirmed by assessing on this genomic region from multiple strains or wild-caught individuals.

## Materials and Methods

### *Drosophila gunungcola* Strain

The *D. gunungcola* SK strain was acquired from P. Wittkopp lab in 2016. Originally established from several females collected in Sukarami, Indonesia (Ishii et al. 2002), this SK strain went through a considerable degree, though not strictly, of inbreeding to reduce the heterozygosity within the genome. This strain was also used to generate D_gunungcola_WGS_1.0; GeneBank: GCA_011057485.1 by Massey et al. (2020).

### DNA and RNA Isolation and Sequencing

High-molecular weight genomic DNA was isolated from the freshly dissected thorax of many 5-day-old males to reduce the contamination of microbial genomes from the digestive tracts. A library of ∼15 kb was prepared and 10 h of diffusion (3–5 Gb) sequencing was performed in three SMRT cells in a PacBio Sequel sequencer in the NGS high-throughput genomics Core in Biodiversity Research Center at Academia Sinica.

RNA sequencing data were acquired from five total RNA samples extracted from pooled individuals of 1) 5 day-old males, 2) 5 day-old females, 3) male pupa at various stages, 4) female pupa at various stages, and 5) larvae at various stages. The poly-A mRNA-based library preparation, using the VAHTS mRNA-seq v3 library preparation kit and VAHTS mRNA Capture Beads, and 150 cycles of pair-end sequencing were performed by “Yourgene” (www.yourgene-health.com). See Supplementary material for a more detailed description and supplementary table S5, Supplementary Material online, for the data statistics.

### Genome De Novo Assembly

A brief workflow of the genome de novo assembly is illustrated in supplementary figure S7, Supplementary Material online, and a more detailed description is available in Supplementary material. A total of 10,760,334,498 bases in 1,190,891 filtered subreads with an average of 9,020 bases were acquired after the quality assessment, and filtering of the raw sequence data was performed using SequelQC v1.3.0 (Hufnagel et al. 2020). The statistics of PacBio reads and QC are summarized in supplementary table S6, Supplementary Material online. These subreads were subjected to generate assemblies independently in Falcon v1.1.5 and Canu v1.8, followed by two rounds of polishing by Arrow (Chin et al. 2013, 2016; Koren et al. 2017). We also performed a hybrid assembly using PacBio-filtered subread and ∼20× Illumina (Massey et al. 2020, GenBank assembly accession: GCA_011057485.1), but the obtained assembly exhibits low contiguity and high assembly error (supplementary table S7, Supplementary Material online). All assemblers were run with default or recommended optimized parameters (available online: https://github.com/AteeshaNegi/dgunungcola). The assembled contigs obtained from three rounds of merging processes in Quickmerge v0.3 were assessed for the presence of bacterial contigs by BUSCO V4.0.5, Kraken2 v2.0.9-beta, and BLAST (Altschul et al. 1997; Simão et al. 2015; Chakraborty et al. 2016; Wood et al. 2019). The final merged assembly was processed in the purge_haplotigs v1.1.1 and inspected by BUSCO using Diptera dataset in OrthoDB v10 (Roach et al. 2018; Kriventseva et al. 2019).

For assigning the contigs to the corresponding Muller elements, a total of 351 primary and repetitive-sequence-masked contigs were aligned against *D. elegans* (assembly accession: GCA_011057505.1) and *D. melanogaster* (r6.36 FlyBase). Based on the sequence comparison to *D. melanogaster*, one 17,498 bp full-length mitochondria genome being flanked by two partial mitochondria sequences was identified in a 46,961-bp contig in the final assembly (supplementary fig. S8*A*, Supplementary Material online). This mitochondria genome was annotated by MITO-S and also manually inspected with the *D. melanogaster* mitochondria genome (supplementary fig. S8*B*, Supplementary Material online).

The quality of the current assembled *D. gunungcola* genome, including completeness, degree of fragmentation, co-linearity, and synteny, was assessed or visualized in BUSCO V4.0.5, Quast package v5.02, IGV v2.11.1, SyMAP v5.0.6, and SyRI v1.5 (Soderlund et al. 2011; Gurevich et al. 2013; Thorvaldsdóttir et al. 2013; Simão et al. 2015; Goel et al. 2019). See Supplementary material for a more detailed description.

### Nuclear Genome Annotation

RNA transcripts were assembled de novo with trimmed and filtered RNA-seq reads on an individual-sample basis by using Trinity v2.8.5 (Haas et al. 2013). The repetitive sequences were masked from the assembled transcripts by RepeatMasker v4.1.0 (Chen 2004). Subsequently, the transcript and gene model were annotated in both transcriptome-based and homolog-based approaches by MAKER v3.01.03 analysis pipeline (Cantarel et al. 2008). We performed an initial and four iterative rounds of MAKER analysis. The best gene model was acquired in the third round, resulting in 96.8% of our gene models bearing an AED of less than 0.5 (supplementary table S8, Supplementary Material online). Subsequently, 13,950 genes with an average gene length of 4,248 in the *D. gunungcola* genome were identified and assessed by BUSCO v4.0.5 against the Diptera dataset (number of BUSCOs = 3285, OrthoDB v10). In total, 10,930 genes were found to be putative orthologs of *D. melanogast*er genes (https://github.com/AteeshaNegi/dgunungcola) by OrthoFinder v2.2.7 (Emms and Kelly 2019). See Supplementary material for a more detailed description.

### Identification of OR and IR Orthologs

The ORs and IRs in *D. gunungcola* and eight closely related *Drosophila* species were identified using protein sequences from *D. melanogaster* in HMMER v.3.3.2 (Eddy 2011). The possible pseudogenes were identified by using PseudoPipe (Zhang et al. 2006). If a gene was not identified from our assembled *D. gunungcola* genome, the protein sequences from our de novo-assembled transcripts were inspected using TransDecoder v5.5.0 (B. Haas and Papanicolaou 2016). The genome information of eight *Drosophila* species is available in Supplementary material.

## Supplementary Material

evad048_Supplementary_Data

## Data Availability

Assembled sequences of the *D. gunungcola* genome have been deposited in the NCBI database (accession number: GCA_025200985.1 for nuclear genome and GenBank sequence CM045947.1 for mitochondrial genome; WGP: JAMKOV01). The raw reads are available as SRA under the NCBI database BioProject PRJNA837195 (or BioSample SAMN28191681).
