# Supplementary material for: Long-read-based Genome Assembly of Drosophila gunungcola Reveals Fewer Chemosensory Genes in Flower-breeding Species
Source: Genome Biol Evol. 2023 Mar 17;15(3):evad048. doi: 10.1093/gbe/evad048 (PMC10063221; doi:10.1093/gbe/evad048)
Supplement: evad048_Supplementary_Data [file evad048_supplementary_data.pdf]

Supplementary Materials For

Title:
Long-read-based genome assembly of *Drosophila gunungcola* reveals fewer chemosensory genes in flower-breeding species

Authors:
Ateesha Negi[1], Ben-Yang Liao[2], and Shu-Dan Yeh[1]*

Affiliation:
[1]Department of Life Sciences, National Central University, Taiwan (R.O.C.)
[2] Division of Biostatistics and Bioinformatics, National Health Research Institutes, Taiwan (R.O.C.)

*Author for Correspondence:
Shu-Dan Yeh, Department of Life Sciences, National Central University, Taiwan (R.O.C.), Tel: 886-3-4227151 ext. 65082, Fax: 886-3-4228482, Email: shudanyeh@ncu.edu.tw

Contents:

Supplemental Materials and Methods:	Pages 1 to 7
Fig. S1-S8:	Pages 8 to 19
Table S1-S8:	Pages 20 to 28

Supplemental Materials and Methods
***Drosophila gunungcola* strain**

The SK strain of *D. gunungcola* used in this study was acquired from P. Wittkopp lab in 2016. This strain was originally established from several females collected in Sukarami, Indonesia (Ishii *et al.*, 2002), was generously provided by Dr. M. T. Kimura to J. True at Stony Brook University, and was subsequently distributed to P. Wittkopp lab at the University of Michigan, Ann Arbor. The SK strain was maintained on a standard cornmeal medium at 23-24°C and went through a considerable degree, though not strictly, of inbreeding to reduce the heterozygosity within the genome.

DNA and RNA isolation and sequencing

High molecular weight genomic DNA was isolated from the freshly dissected thorax of many 5-day-old males to reduce the contamination of microbial genomes from the digestive tracts. Approximately 65 µg of DNA was extracted using Zymo Quick DNA-Insect Midiprep plus Kit (catalog number D4075). A library of ~15 kb was prepared and 10 hours of diffusion (3-5GB) sequencing was performed in three SMRT cells in a PacBio Sequel sequencer in the NGS high throughput genomics Core in Biodiversity Research Center at Academia Sinica.

RNA-sequencing data were acquired from five total RNA samples extracted from pooled individuals of 1) 5 day- old males, 2) 5 day- old females, 3) male pupa at various stages, 4) female pupa at various stages, and 5) larvae at various stages. Flies were snapped frozen by liquid nitrogen in 1.5ml microcentrifuge tubes and kept at -80 °C. The total RNAs were extracted using Quick-RNA MiniPrep kit (catalog number R1054; Zymo Research) and were examined by Qbit fluorometer (RNA HS assay kit, ThermoFisher Scientific) and gel electrophoresis analysis in 2% agarose gel before shipped to Yourgene (www.yourgene-health.com) where library preparation and sequencing were performed. The quality of total RNAs was inspected by Nano-300 Micro-Spectrophotometer in Yourgene before the library preparation. The libraries with ~410 bp inserts, prepared using VAHTS mRNA Capture Beads and VAHTS Universal v3 RNA-seq Library Prep kit for Illumina, were barcoded and subjected to 150 cycles of pair-end sequencing in NovaSeq (Illumina sequencing platform). An average of ~90 million raw reads were acquired per sample. See Table S5 for the data statistics.

Genome *de novo* assembly

Brief workflow of the genome *de novo* assembly is illustrated in Supplemental Fig. S6. A total of 10,760,334,498 bases in 1,190,891 filtered subreads with an average of 9,020 bases was acquired after the quality assessment and filtering of the raw sequence data was performed using SequelQC v1.3.0 (Hufnagel et al., 2020). The statistics of PacBio reads and QC are summarized in Supplemental Table S6. These subreads were subjected to generate assemblies independently in Falcon v1.1.5 and Canu v1.8, followed by two rounds of polishing by Arrow (Chin et al., 2013; Chin et al., 2016; Koren et al., 2017). We also performed a hybrid assembly using PacBio filtered subread and ~20x Illumina reads previously generated from the same strain (Massey et al., 2020; GenBank assembly accession: GCA_011057485.1) in the hybrid assembler MaSuRCA v3.2.2 (Zimin et al., 2013), followed by two rounds of polishing in Pilon v1.22 (Walker et al., 2014). This hybrid assembly was not proceeded further, owing to low contiguity and high assembly error as assessed by BUSCO (Supplemental Table S7). All assemblers were run with default or recommended optimized parameters available online at https://github.com/AteeshaNegi/dgunungcola. The assembly of 212 Mb in 676 contigs with N50 of 2,135,404 bases and the longest contig of 31,037,916 bases was obtained from three rounds of merging processes in Quickmerge v0.3 (Chakraborty et al., 2016). These three round merging includes 1) two original assemblies from each long-read assembler, 2) Canu assembly and merged *assembly 1* acquired from the first round, and 3) Falcon assembly and merged *assembly 2* acquired from the second round as well as the following two polishing rounds in Arrow (Chin et al., 2013).

One of the assembled contigs consists of mitochondria sequences (see the section below), thus was excluded from the further processes of removing contaminated sequences. The processes include 1) the examination of the standard contaminant domain in Kraken2 V2.0.9-beta (Wood et al., 2019), 2) the scan of any bacterial contigs using Bacteria dataset from OrthoDB v10 in BUSCO v4.0.5 (Simão et al., 2015), and 3) the BLAST search of sequences, downloaded from NCBI, of the *Drosophila* gut microbiome, listed in Broderick and Lemaitre 2012, and yeast genome with sequences. To account for all haplotypes, the final merged assembly was processed with recommended pipeline in the purge_haplotigs v1.1.1 (Roach et al., 2018). The presence of duplicated haplotypes was also inspected by duplicated BUSCO v4.0.5 along with the Diptera dataset (use '*-l diptera_odb10*' command) in OrthoDB v10 (Simão et al., 2015; Kriventseva et al., 2019). After haplotype and contaminant sequence removal (21.8 Mb in 302 contigs and 0.39 Mb

in 23 contigs, respectively), our final assembly consists of 351 primary contigs with an N50 of 3,022,255 bases and the longest contig of 31,037,916 bases.

The respective Muller elements of 351primary contigs were determined by aligning against *D. elegans* (GCA_011057505.1) and *D. melanogaster* (r6.36 FlyBase). The repetitive sequences of the compared genomes were masked by RepeatMasker v4.1.0 (Chen, 2004) using the available information from Repbase (Bao et al., 2015) to avoid the misalignments. The pairwise alignments of the whole-genome sequence were carried out by NUCmer using *'--maxmatch -c 100 -b 500 -l 50'* in the MUMmer v3.1(Kurtz et al., 2004). The best mapping of contigs to the reference was determined by delta-filter utility of show-cord programs in MUMmer. The contigs were arranged into chromosomal-level scaffolds using show-tiling programs in MUMmer. The unalignable contigs were grouped into unclassified contigs. We did not intend to stitch contigs in order to prevent introducing artifacts of structural variation in the following analyses of synteny.

Several methods were employed to assess the quality of the current assembled *D. gunungcola* genome. First, this assembly's completeness and sequence accuracy was inspected in BUSCO v4.0.5 with the Diptera dataset (use '*-l diptera_odb10*' command) in OrthoDB v10 (BUSCOs = 3,285) (Simão et al., 2015; Kriventseva et al., 2019). Second, the degree of fragmentation in this assembly fragmentation was evaluated by Quast package v5.02 (Gurevich et al., 2013), in which the standard assembly statistics (average contig size, number of contigs, assembled genome size, N50, etc) were calculated. Third, the co-linearity between the raw reads and the first ten longest contigs was indexed and aligned using pbmm2 (https://github.com/PacificBiosciences/pbmm2) and then incepted via visualized on IGV v2.11.1 (Thorvaldsdóttir et al., 2013). Finally, the synteny for chromosome arm-level conservation between *D. gunungcola* and its related species was examined in SyMAP v5.0.6 (Soderlund et al., 2011).

Mitogenome genome identification and annotation

A 46,961-bp contig consisting of mitochondrial genome sequence was identified from the final assembly (see nuclear genome assembly section) by the BLASTn (Altschul, et al., 1997) search with *D. melanogaster* mitochondrial genome (NCBI assession: NC_024511.2). Eight subreads from our PacBio data correspond to the assembly of this contig. This contig presumably includes one 17,498 bp full-length mitochondria genome, flanked by two partial mitochondria

sequences, 16,268 and 13,036 bp, respectively (Supplemental Fig. S7). The annotation of the *D. gunungcola* genome was done by MITOS-2 using the "*metazoan*" reference option and the "*invertebrate*" genetic code (Donath et al., 2019). By using *D. melanogaster* mitogenome as a reference, the *D. gunungcola* mitogenome was also manually inspected and annotated within SnapGene® software v5.3.2 (Insightful Science).

Genome annotation

RNA-seq reads with a quality lower than Q30 were filtered out either after an extra trimming step from the adult female sample or directly from the rest four samples in Fastp v0.19.1 (Shifu et al., 2018; Table S5). To minimize the artifacts of gene fusion in high-density regions, the *de novo* assembly of the transcriptome was performed on an individual-sample basis by using Trinity v2.8.5 (Haas et al., 2013) with the '*jaccard*_clip' option. Before annotating the gene model, the transposable elements (TEs) were identified and masked from *D. gunungcola* genome sequences using RepeatMasker v4.1.0 and the combined library of repetitive sequences retrieved from Dfam_3.1 and Repbase using RepeatModeler v1.0.11 (Chen, 2004; Smit & Hubley, 2008). The transcript and gene models were annotated by following the MAKER v3.01.03 analysis pipeline (Cantarel et al., 2008), in which both transcriptome-based and homolog-based approaches were carried out. The initial MAKER analysis was executed with five main data files, including 1) *de novo* assembled *D. gunungcola* genome, 2) *de novo* assembled transcriptome from RNA-seq data, 3) a full dataset of protein sequences from eight *Drosophila* species, including *D. ananassae* r1.3, *D. erecta* r1.3, *D. melanogaster* r6.36, *D. sechellia* r1.3, *D. simulans* r2.02, and *D. yakuba* r1.03 obtained from FlyBase (https://flybase.org/), and *D. elegans* and *D. suzukii* obtained from NCBI (GenBank with assembly accession number: GCA_018152815.1 and GCF_013340165.1, respectively), 4) a combined library of repetitive sequences in *D. gunungcola*, and 5) protein sequences of TE for annotating TE-related proteins. The gene models curated from the initial round were used to train *ab initio* gene predictors software, Augustus and SNAP, and simultaneously evaluated by BUSCO analysis using the Diptera dataset (*'-l diptera_odb10*' command, BUSCOs = 3,285) in OrthoDB v10. The subsequent four MAKER rounds were iteratively run with these trained *ab initio* gene predictors. The best gene model was acquired in the third round of MAKER annotation as suggested by the BUSCO score and annotation edit distance distribution (Supplemental Table S7). Overall, 96.8% of our gene models bear less than 0.5 or better AED. Using the gene model of eight closely-related species, 14,265 genes and 14,703 transcripts (with

isoforms derived from some genes) were predicted in the *D. gunungcola* genome. But only 13,950 protein-coding genes with an average gene length of 4,248 bp were supported by our RNA-seq data. In total, 10,930 genes were found to be putative orthologs of *D. melanogast*er genes (https://github.com/AteeshaNegi/dgunungcola) via OrthoFinder v2.2.7 (Emms & Kelly, 2019). These results suggest that most of our annotations are of good quality.

Identification of ORs and IRs orthologs

The ORs and IRs in *D. gunungcola* and eight closely-related *Drosophila* species, including *D. elegans* (GCF_018152505.1), *D. ananassae* (GCF_017639315.1), *D. suzukii* (GCF_013340165.1), *D. yakuba* (GCF_016746365.2), *D. erecta* (GCF_003286155.1), *D. simulans* (GCF_016746395.2), *D. sechellia* (GCF_004382195.2), and *D. melanogaster* (FlyBase r6.36), were identified using protein sequences from *D. melanogaster* retrieved from Pfam database (7tm_6 or PF02949 for ORs; Lig_chan and Lig_chan-Glu_bd or PF00060 and PF10613 for IRs) in HMMER (Eddy 2011). The longest protein isoform was selected when multiple isoforms were found. If a gene was not identified from our assembled *D. gunungcola* genome, the protein sequences from our *de novo* assembled transcripts were inspected using TransDecoder v5.5.0 (B. Haas & Papanicolaou, 2016). We further identified IRs and ORs pseudogenes from the *D. gunungcola* genome using PseudoPipe (Zhang et al., 2006). The top hits of HMMER results from the peptide sequences for above mentioned eight closely-related *Drosophila* species and all ORs and IRs CDS of *D. gunungcola* and *D. elegans* are available online at https://github.com/AteeshaNegi/dgunungcola.

Data visualization

We used several tools and software were used to visualized the data. The plots of contig alignments were generated by importing MUMmer output files to SyMAP v5.0.6 and SyRI v1.5 (Soderlund et al., 2011; Goel et al., 2019). The sequence alignment and gene model were visualized in IGV v2.11.1 (Thorvaldsdóttir et al., 2013).

References:

Altschul, S. F., Madden, T. L., Schäffer, A. A., Zhang, J., Zhang, Z., Miller, W., & Lipman, D. J. (1997). Gapped BLAST and PSI-BLAST: a new generation of protein database search programs. *Nucleic Acids Research, 25*(17), 3389-3402.

Broderick NA, Lemaitre B. 2012. Gut-associated microbes of *Drosophila melanogaster*. *Gut Microbes* 3(4):307-321.

Cantarel, B. L., Korf, I., Robb, S. M., Parra, G., Ross, E., Moore, B., et al. (2008). MAKER: an easy-to-use annotation pipeline designed for emerging model organism genomes. *Genome research, 18*(1), 188-196.

Chakraborty, M., Baldwin-Brown, J. G., Long, A. D., & Emerson, J. J. (2016). Contiguous and accurate de novo assembly of metazoan genomes with modest long read coverage. *Nucleic Acids Research, 44*(19), e147-e147.

Chen, N. (2004). Using Repeat Masker to identify repetitive elements in genomic sequences. *Current protocols in bioinformatics, 5*(1), 4.10. 11-14.10. 14.

Chen, S., Zhou, Y., Chen, Y., & Gu, J. (2018). fastp: an ultra-fast all-in-one FASTQ preprocessor. Bioinformatics, 34(17), i884-i890.

Chin, C.-S., Alexander, D. H., Marks, P., Klammer, A. A., Drake, J., Heiner, C., et al. (2013). Nonhybrid, finished microbial genome assemblies from long-read SMRT sequencing data. *Nature methods, 10*(6), 563-569.

Chin, C.-S., Peluso, P., Sedlazeck, F. J., Nattestad, M., Concepcion, G. T., Clum, A., et al. (2016). Phased diploid genome assembly with single-molecule real-time sequencing. *Nature methods, 13*(12), 1050-1054.

Donath, A., Jühling, F., Al-Arab, M., Bernhart, S. H., Reinhardt, F., Stadler, P. F., et al. (2019). Improved annotation of protein-coding genes boundaries in metazoan mitochondrial genomes. *Nucleic Acids Research, 47*(20), 10543-10552.

Eddy, S. R. (2011). Accelerated profile HMM searches. *PLoS computational biology*, *7*(10), e1002195.

Emms, D. M., & Kelly, S. (2019). OrthoFinder: phylogenetic orthology inference for comparative genomics. *Genome biology, 20*(1), 1-14.

Goel, M., Sun, H., Jiao, W.-B., & Schneeberger, K. (2019). SyRI: finding genomic rearrangements and local sequence differences from whole-genome assemblies. *Genome biology*, 20(1), 1-13.

Gurevich, A., Saveliev, V., Vyahhi, N., & Tesler, G. (2013). QUAST: quality assessment tool for genome assemblies. *Bioinformatics, 29*(8), 1072-1075.

Haas, B., & Papanicolaou, A. (2016). TransDecoder (find coding regions within transcripts). *GitHub 3.1*.

Haas, B. J., Papanicolaou, A., Yassour, M., Grabherr, M., Blood, P. D., Bowden, J., et al. (2013). De novo transcript sequence reconstruction from RNA-seq using the Trinity platform for reference generation and analysis. *Nature protocols, 8*(8), 1494-1512.

Hufnagel, D. E., Hufford, M. B., & Seetharam, A. S. (2020). SequelTools: a suite of tools for working with PacBio Sequel raw sequence data. *BMC Bioinformatics, 21*(1), 429.

Koren, S., Walenz, B. P., Berlin, K., Miller, J. R., Bergman, N. H., & Phillippy, A. M. (2017). Canu: scalable and accurate long-read assembly via adaptive k-mer weighting and repeat separation. *Genome research, 27*(5), 722-736.

Kriventseva EV, et al. 2019. OrthoDB v10: sampling the diversity of animal, plant, fungal, protist, bacterial and viral genomes for evolutionary and functional annotations of orthologs. *Nucleic Acids Res.* 47(D1), D807–D811.

Kurtz, S., Phillippy, A., Delcher, A. L., Smoot, M., Shumway, M., Antonescu, C., & Salzberg, S. L. (2004). Versatile and open software for comparing large genomes. *Genome biology, 5*(2), 1-9.

Roach, M. J., Schmidt, S. A., & Borneman, A. R. (2018). Purge Haplotigs: allelic contig reassignment for third-gen diploid genome assemblies. *BMC Bioinformatics, 19*(1), 1-10.

Simão, F. A., Waterhouse, R. M., Ioannidis, P., Kriventseva, E. V., & Zdobnov, E. M. (2015). BUSCO: assessing genome assembly and annotation completeness with single-copy orthologs. *Bioinformatics, 31*(19), 3210-3212.

Soderlund, C., Bomhoff, M., & Nelson, W. M. (2011). SyMAP v3.4: a turnkey synteny system with application to plant genomes. *Nucleic Acids Research, 39*(10), e68-e68.

Thorvaldsdóttir, H., Robinson, J. T., & Mesirov, J. P. (2013). Integrative Genomics Viewer (IGV): high-performance genomics data visualization and exploration. *Briefings in bioinformatics, 14*(2), 178-192.

Walker, B. J., Abeel, T., Shea, T., Priest, M., Abouelliel, A., Sakthikumar, S., et al. (2014). Pilon: an integrated tool for comprehensive microbial variant detection and genome assembly improvement. *PloS one, 9*(11), e112963.

Wood, D. E., Lu, J., & Langmead, B. (2019). Improved metagenomic analysis with Kraken 2. *Genome biology, 20*(1), 1-13.

Zhang, Z., Carriero, N., Zheng, D., Karro, J., Harrison, P. M., & Gerstein, M. (2006). PseudoPipe: an automated pseudogene identification pipeline. Bioinformatics, 22(12), 1437-1439.

Zimin, A. V., Marçais, G., Puiu, D., Roberts, M., Salzberg, S. L., & Yorke, J. A. (2013). The MaSuRCA genome assembler. *Bioinformatics, 29*(21), 2669-2677.

A)

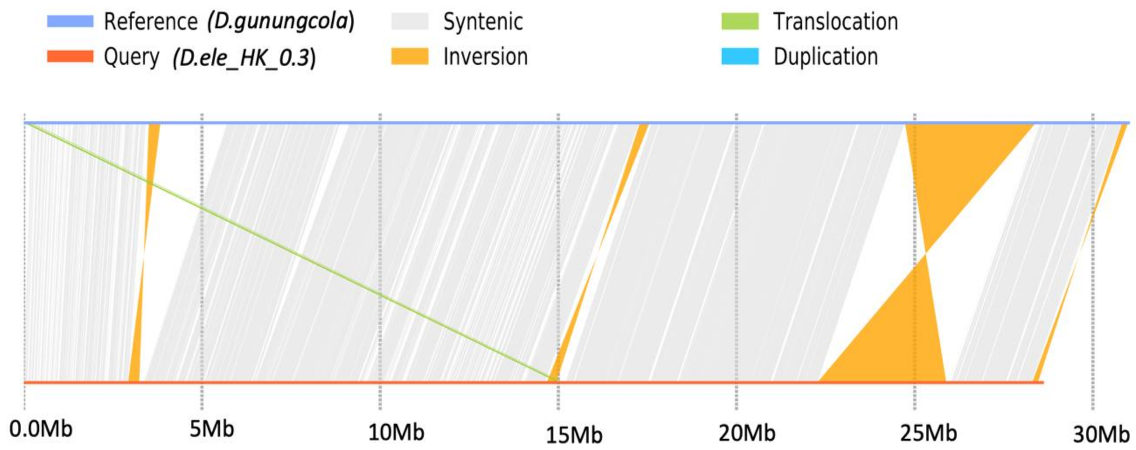

B)

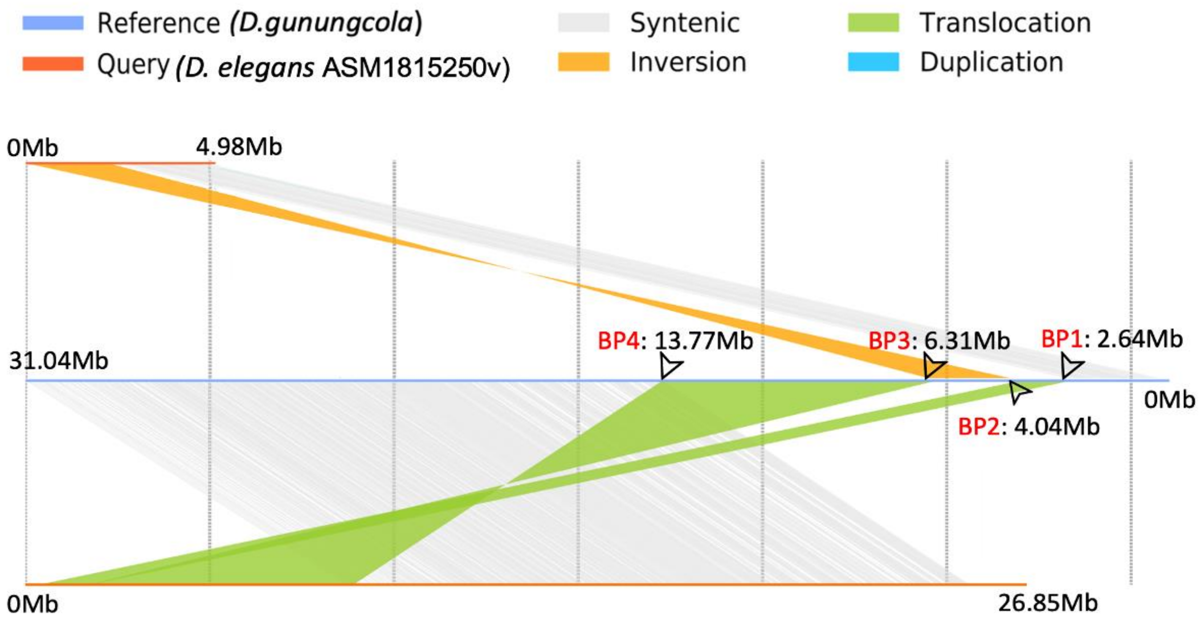

Fig. S1. Comparisons of the largest contig of *D. gunungcola* to Muller element E from two previous assemblies of *D. elegans*. *D. gunungcola* (reported in this study) largest contig (000000F) aligned to A) *D. elegans* assembly (Dele_HK_0.3 short read based; GenBank assembly accession: GCA_018152815.1) and B). *D. elegans* hybrid assembly (ASM1815250v1 long and short read based; GenBank assembly accession: GCA_018152505.1). The plots were generated in SyRI.

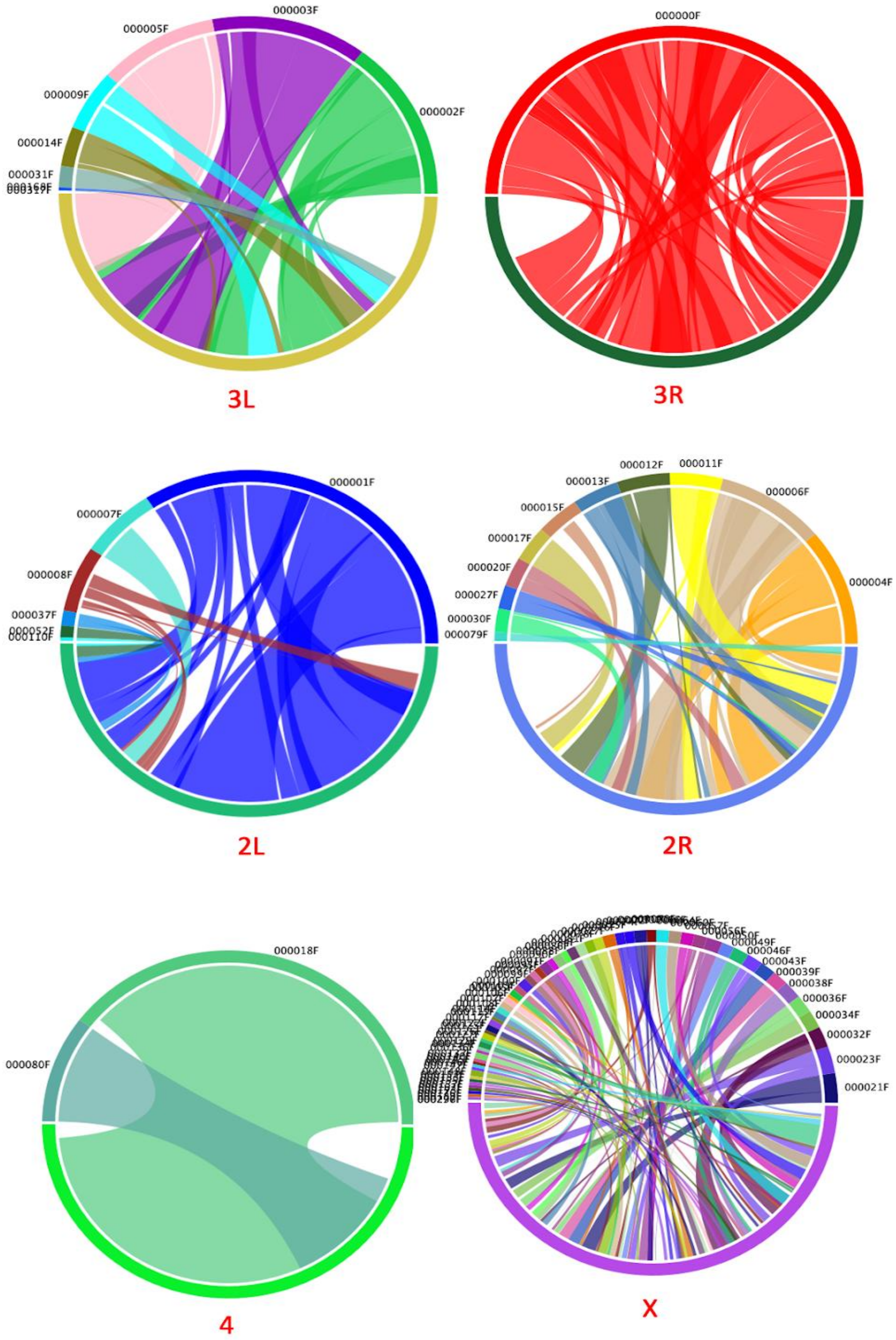

Fig. S2. *D. gunungcola* contigs mapped to each chromosomal arm of *D. melanogaster* r6.36 chromosomes (bottom). A total of 28 contigs were identified that belonged to autosomes and 66 contigs were identified that belong to sex chromosomes. A BLASTp with *D. melanogaster* Y-linked protein was performed and we were able to find eleven types of Y-related genes (out of 25 total genes that are present on y-chr of *D. melanogaster*) with very low sequence similarity <20% mapped to 8 contigs (these contigs are 313F, 201F, 179F, 175F, 162F,121F, 50F, 56F, 49F and 41F they also show approximately 30 % or more sequence similarity to X chr. The graphs were generated using the default settings in SyMAP.

A)

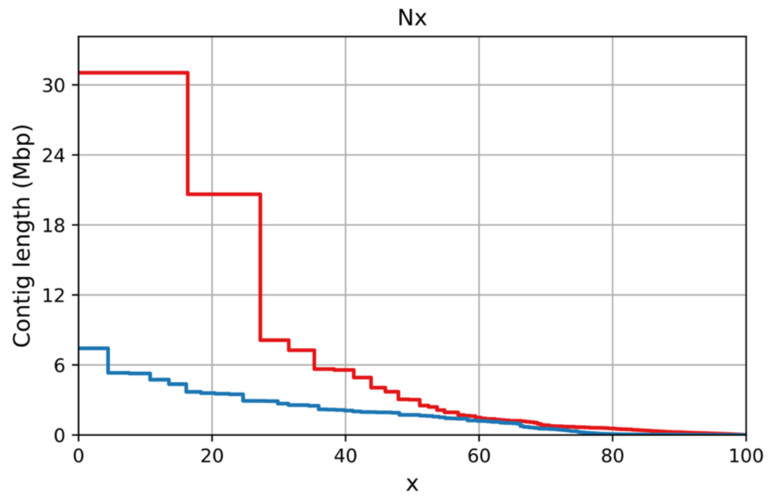

B)

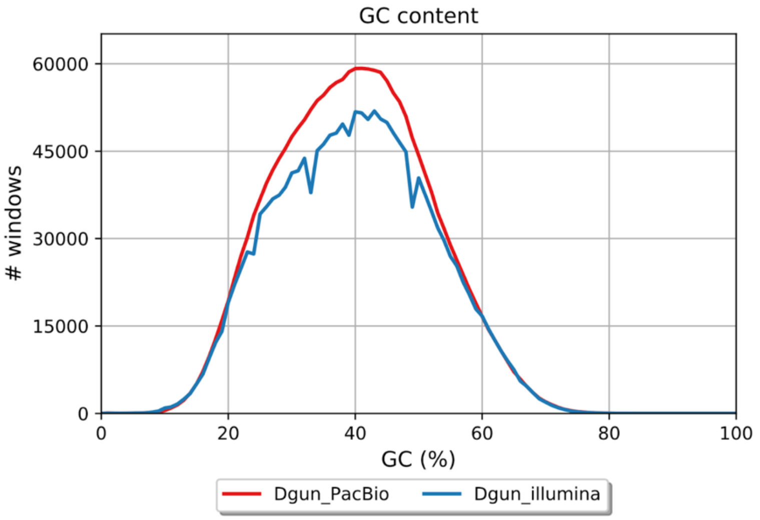

Figure S3. Contiguity and GC % distribution of two assemblies for the *D. gunungcola* genome, the current assembly in red and the previous assembly in blue (GenBank assembly accession: GCA_011057485). (A) Each horizontal section of a line represents a contig with the length, indicated by the *y-axis*, that comprises a certain percentage of the genome, indicated by the *x-axis*. *D. gunungcola* genome assembled in this study (red) represents more than 50% of the genome in less than 15 contigs, indicating high contiguity. (B) GC content distribution of the non-overlapping 100bp windows**.** The distribution of GC content in this assembly exhibits a normal distribution as expected for a single genome. Multiple GC peaks seen in the previous assembly indicate possible sequence contamination. The plots were generated using Quast assembly statistics tool.

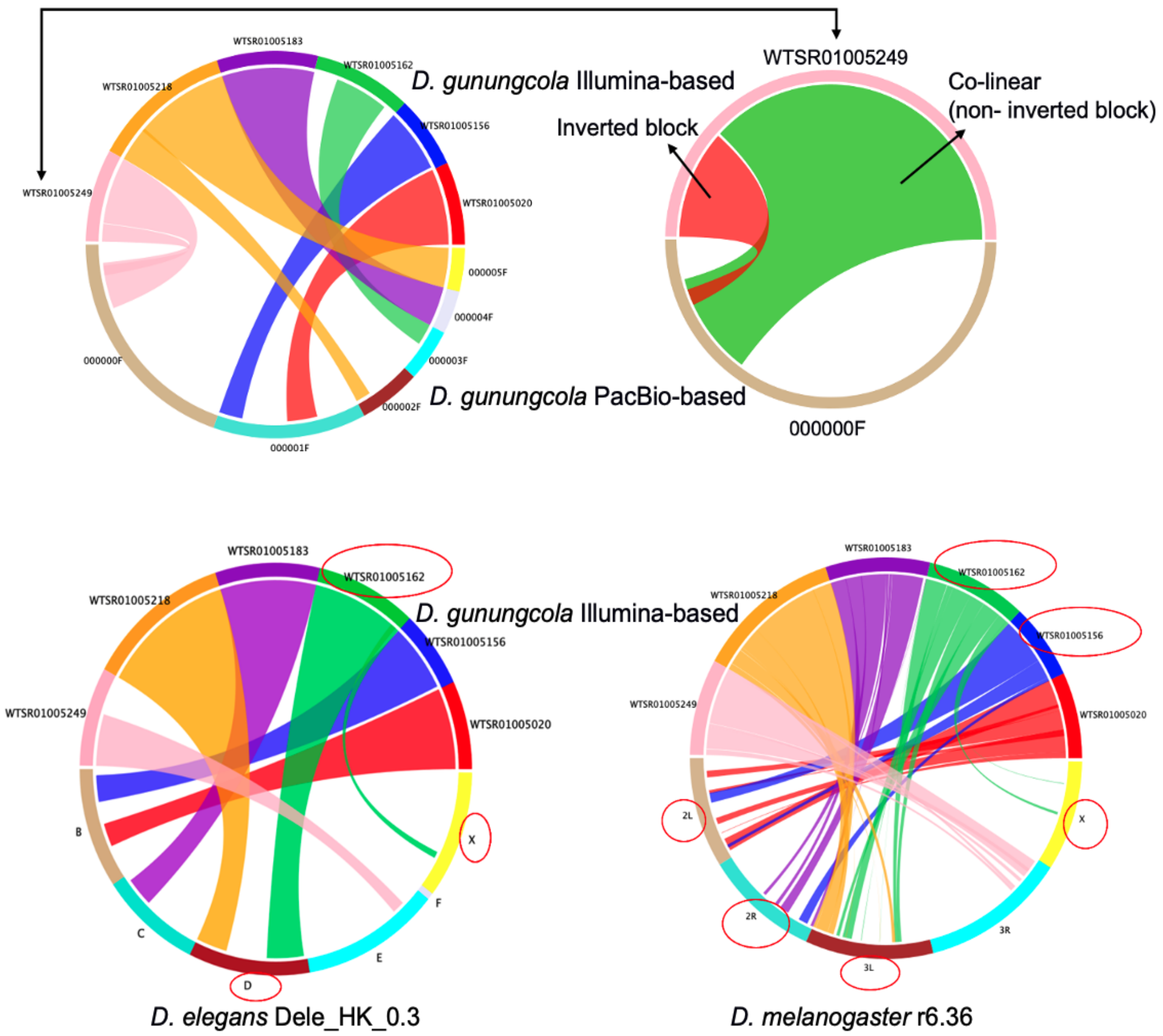

Figure S4. Comparing the previous *D. gunungcola* (Illumina-based) assembly to three assemblies. The six largest scaffolds in *D. gunungcola* Illumina-based (GenBank assembly accession: GCA_011057485.1.) were aligned to *D. gunungcola* PacBio-based assembly obtained in this study, *D. elegans* (GenBank assembly accession: GCA_011057505.1*), and D. melanogaster* r6.36 (FlyBase). This alignment result shows inversions (red color inverted block) and translocations (red circle highlighted scaffolds) probably caused due to contig stitching error in the previous *D. gunungcola* assembly. The graphs were generated using the default settings in SyMap.

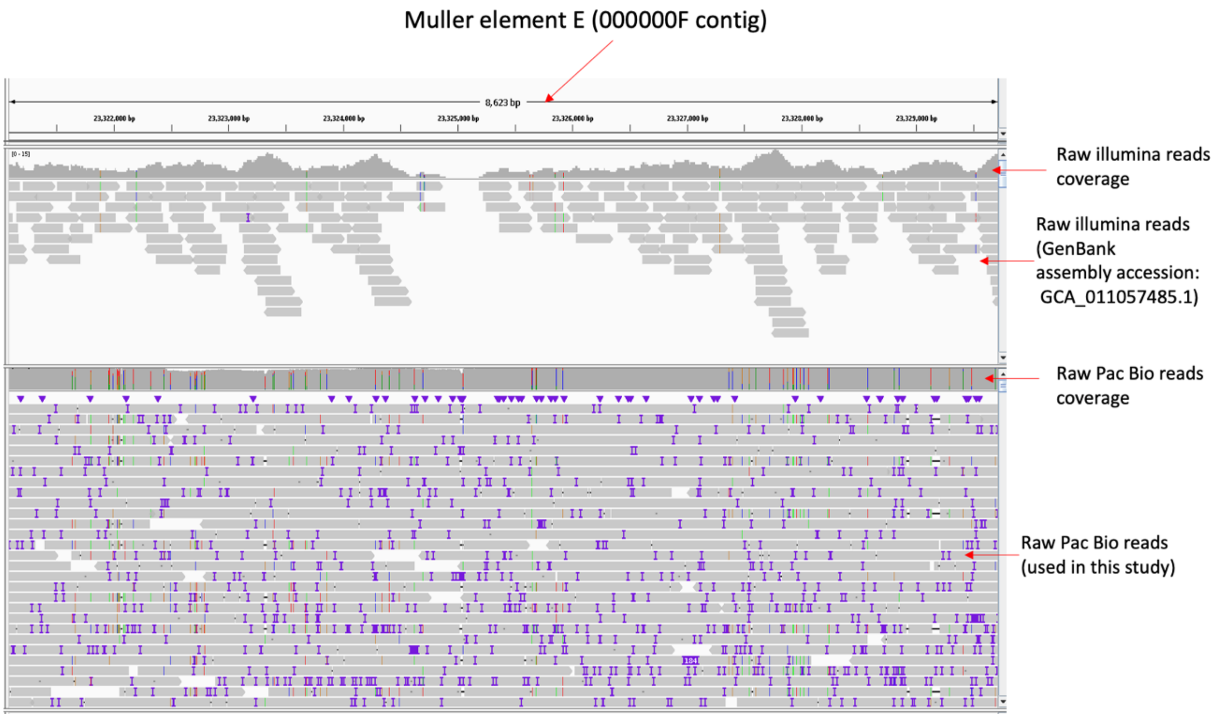

Fig. S5. Raw reads used in two assemblies aligned to our assembled contig, illustrated by the partial region of the largest contig. Raw reads of *D. gunungcola* previous (top window) and this assembly (bottom window) aligned to the largest contig (of this assembly: 000000F breakpoint region of the inverted block indicated in Fig. S4. The uniform coverage rate and highly overlapping PacBio reads indicate a proper assembly. This graph was generated in Integrative Genome Browser.

A)

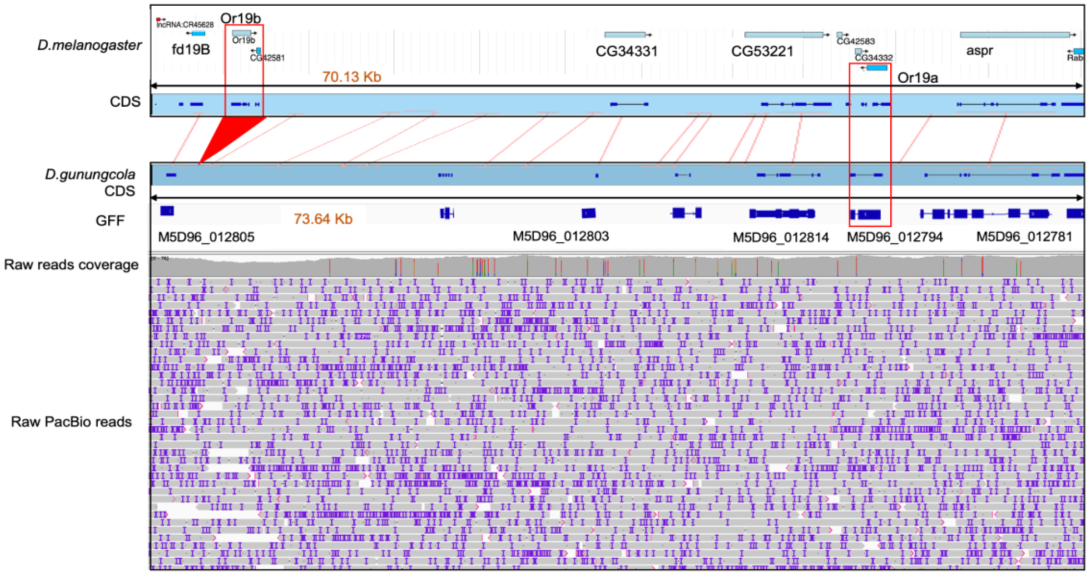

Or19b and Or19a gene region. Protein sequences of *fd19B*, *CG34331*, *CG53221*, *Or19a* and *aspr* of *D. melanogaster* are 65, 44, 94, 69 and 84 percent identical to the protein sequence encoded in M5D96_012805, M5D96_012803, M5D96_012814, M5D96_012794 and M5D96_012781 of *D. gunungcola*. There was no RNA-seq reads mapped to the potential *Or19b* region.

B)

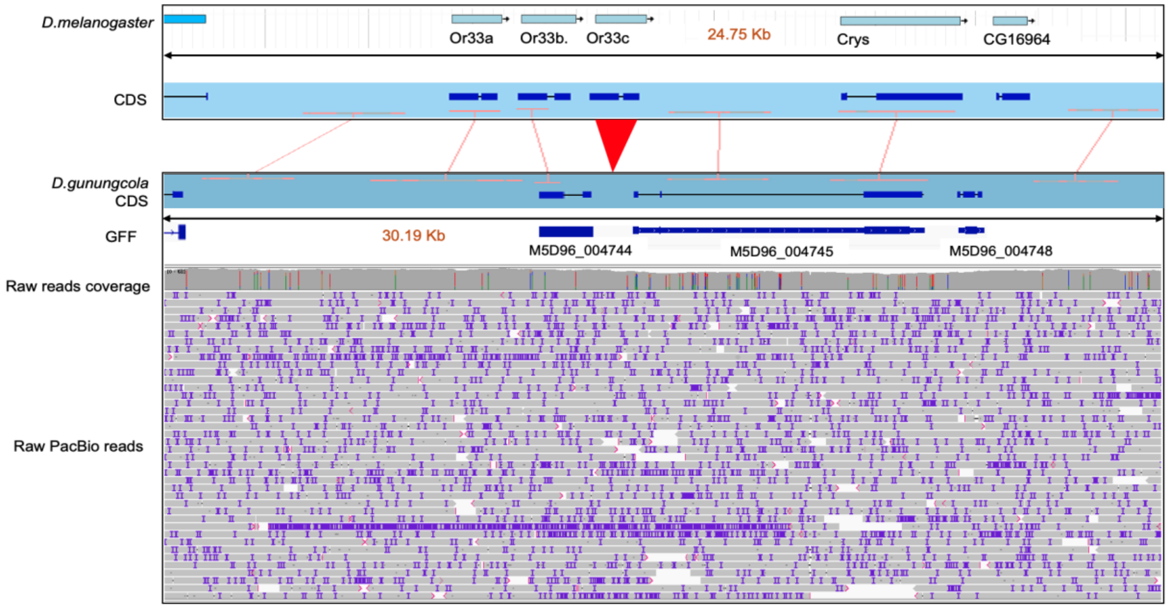

Or33c gene region. Protein sequences of *Or33b* and *Crys* of *D. melanogaster* are 52, and 85 percent identical to the protein sequences encoded in M5D96_004744 and M5D96_004745 of *D. gunungcola*, respectively. *Or33a* and *Or33b* were manually annotated based on RNA-seq reads. *Or33c* was annotated as a pseudogene by PseudoPipe analysis.

D)

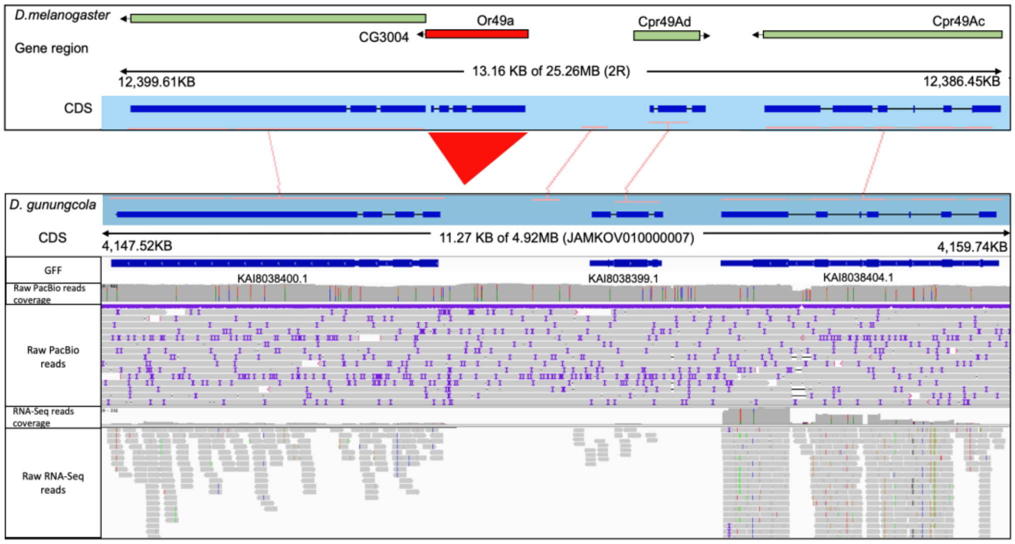

Or49a gene region. Protein sequences of *CG30048*, *Cpr49Ad*, and *Cpr49Ac* of *D. melanogaster* are 51, 90, and 87 percent identical to the protein sequence encoded in KAI8038400.1, KAI8038399.1, and KAI8038404.1, respectively. There was no RNA-seq reads mapped to the potential *Or49a* region.

E)

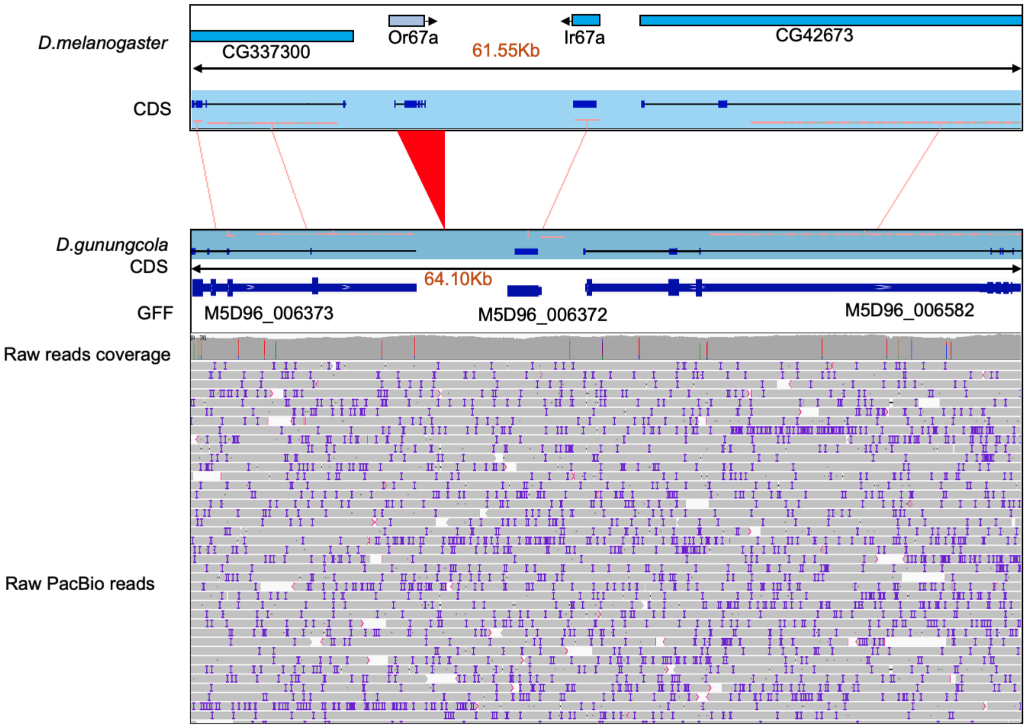

Or67a gene region. Protein sequences of *Ir67a* and *CG33700* of *D. melanogaster* are 77, and 63 percent identical to the protein sequence encoded in M5D96_006372 and M5D96_006373, respectively. There was no RNA-seq reads mapped to the potential *Or67a* region.

F)

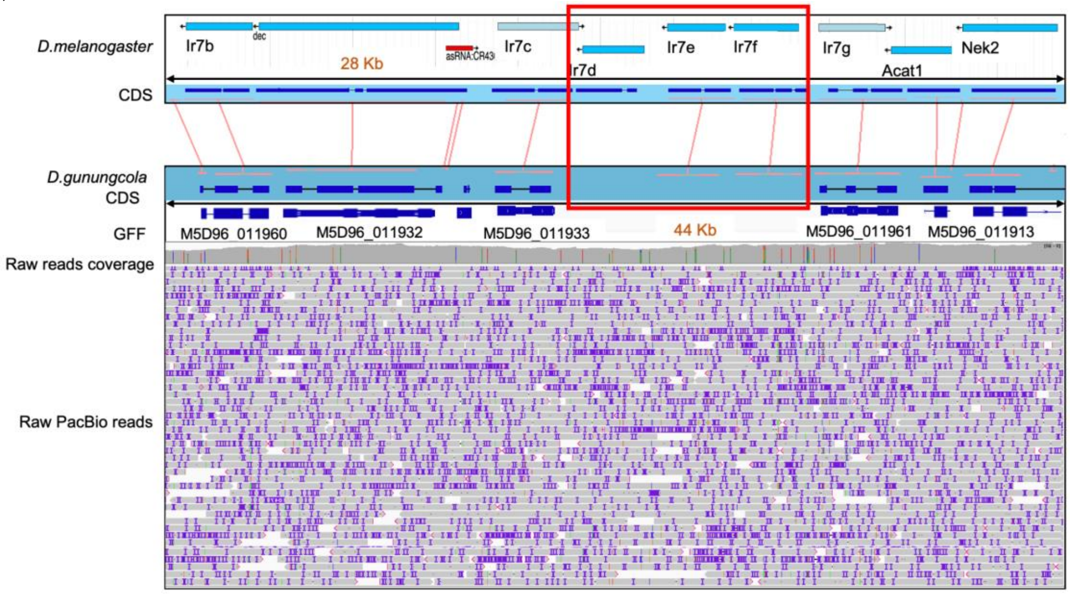

Ir7f gene region. Protein sequences of *Ir7c* and *Ir7g* of *D. melanogaster* are 56 and 61 percent identical to the protein sequence encoded in M5D96_01193 and M5D96_011961 of *D. gunungcola*, respectively. *Ir7d* and *Ir7e* were manually annotated based on RNA-seq reads. *Ir7f* was annotated as a pseudogene by PseudoPipe analysis and its coding sequence contains several premature stop codons.

G)

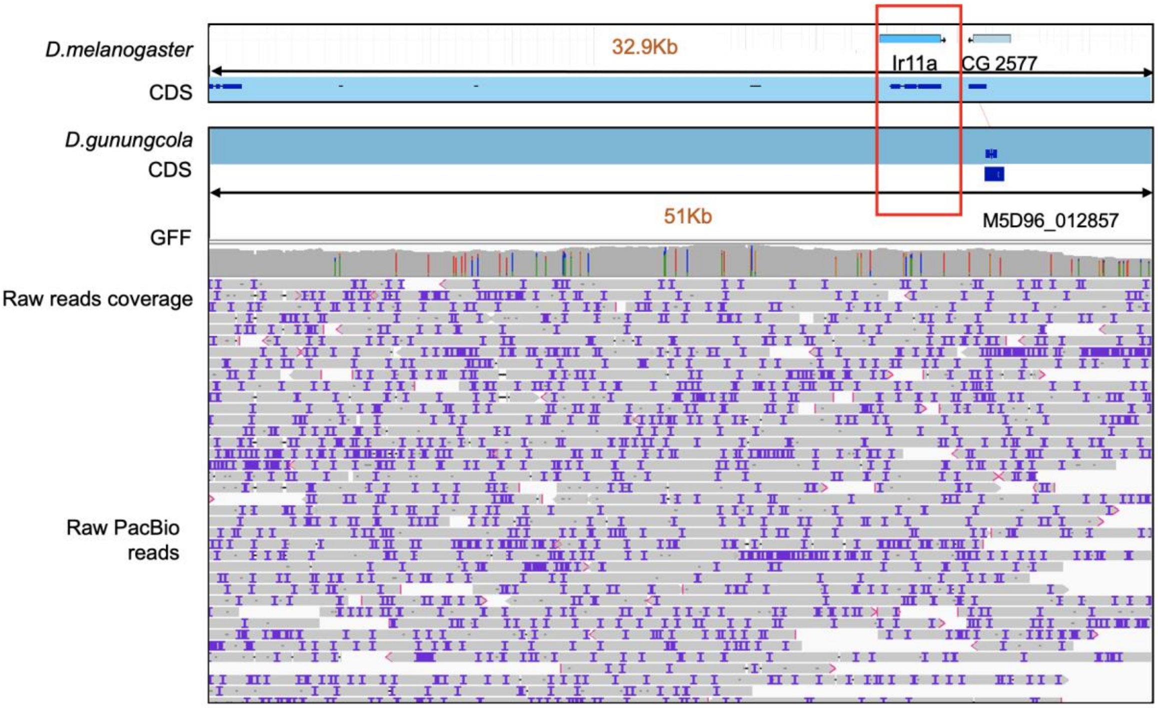

Ir11a gene region. CG2577 protein sequence of *D. melanogaster* is 56 percent identical to the protein sequence encoded in M5D96_012857 of *D. gunungcola*. No RNA-seq reads were mapped to this region.

H)

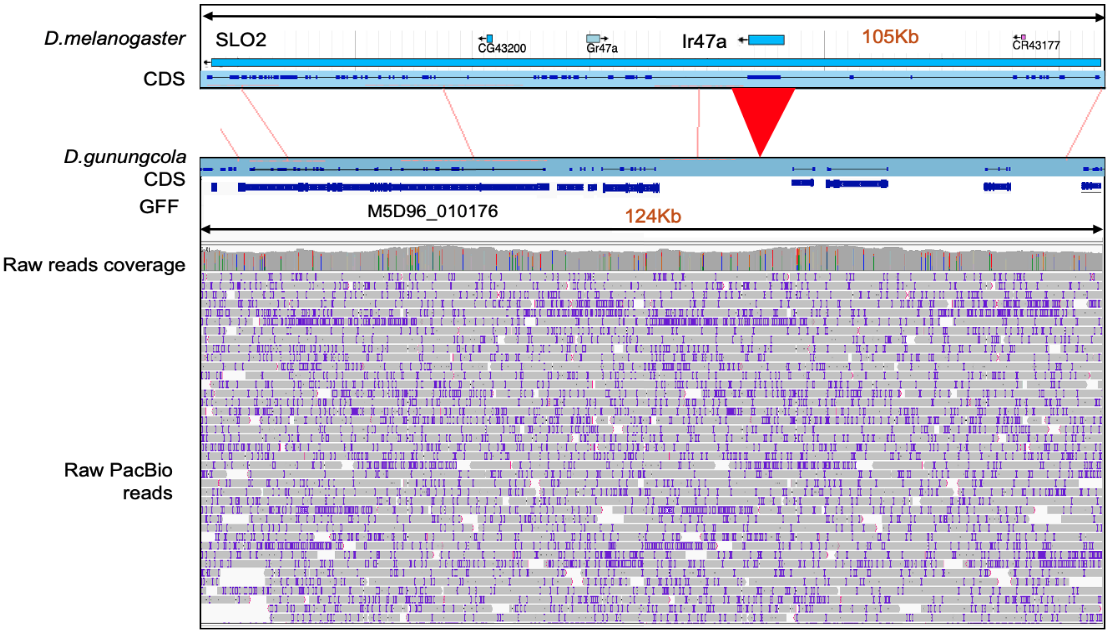

Ir47a gene region. SLO2 protein sequence of *D. melanogaster* is 51 percent identical to the protein sequence encoded in M5D96_010176 of *D. gunungcola*. *Ir47a* was found to be fragmented in PseudoPipe analysis. Several premature stop codons were found in the potential *Ir47a* coding region.

I)

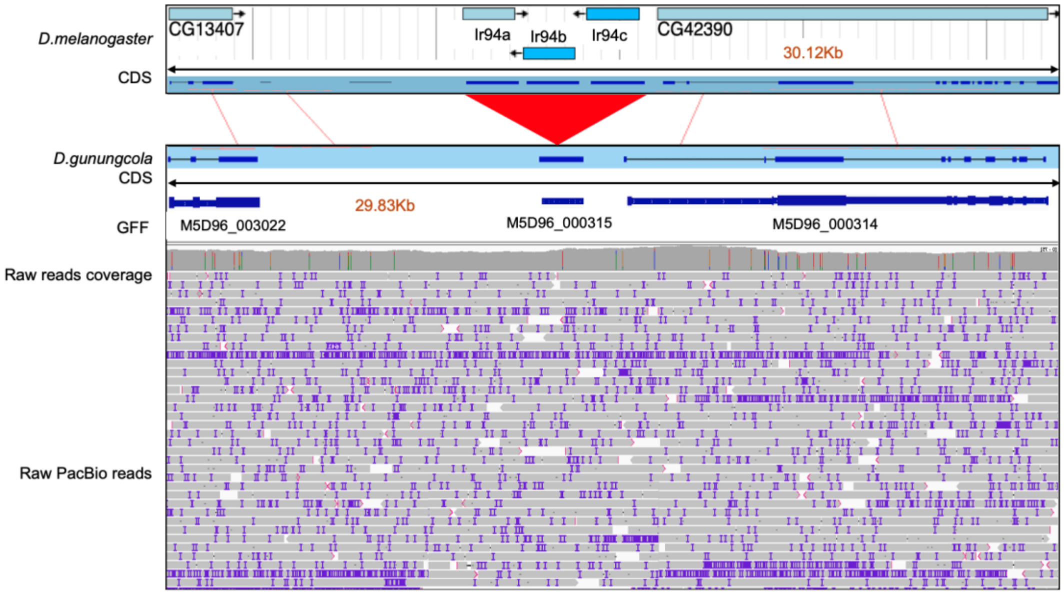

Ir94a/b/c gene region. CG13407 and CG42390 protein sequences of *D. melanogaster* are 92.9 and 70.5 percent identical to the protein sequence encoded in M5D96_003022 and M5D96_000134 of *D. gunungcola*, respectively. The protein sequence encoded in M5D96_000315 are 36.76%, 32.4%, and 31% identical to *Ir94c*, *Ir94b*, and *Ir94a* protein sequences in *D. melanogaster*, respectively. No RNA-seq reads were mapped to M5D96_000315 region.

J)

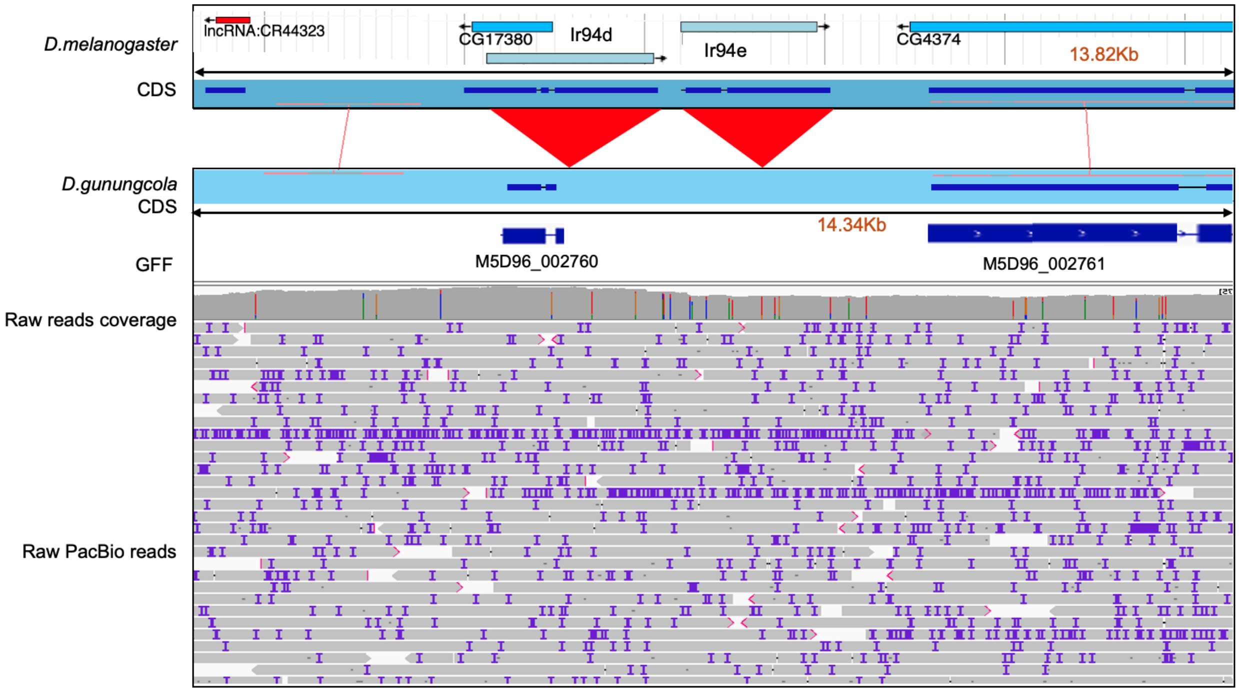

IR94d/e gene region. CG17380 and CG4374 protein sequence of *D. melanogaster* are 55.3 and 86.5 percent identical to the protein sequence encoded in M5D96_002760 and M5D96_002761 of *D. gunungcola*, respectively. Few RNA-seq reads were mapped to the suspected Ir94d and Ir94e regions, in which were annotated as fragmented by PsuedoPipe analysis.

Figure S6 Genomic regions of *D. gunungcola* that show the loss of odorant receptor (OR) and inotropic receptor (IR) genes in comparisons of gene synteny to *D. melanogaster*. In each genomic region, the upper panel shows the region containing OR or IR genes in *D. melanogaster* and the lower panel shows the homologous region, indicated by the orthologs adjacent to OR or IR genes, in *D. gunungcola*. The red inverted triangles mark the OR or IR genes missing in *D. gunungcola*. The uniform coverage rate of raw PacBio read across a genomic region and the raw read extending the adjacent genes support the correct assembly of the region. The graphs were generated in IGV (the lower panel) and SyMAP with the default settings (the upper panel).

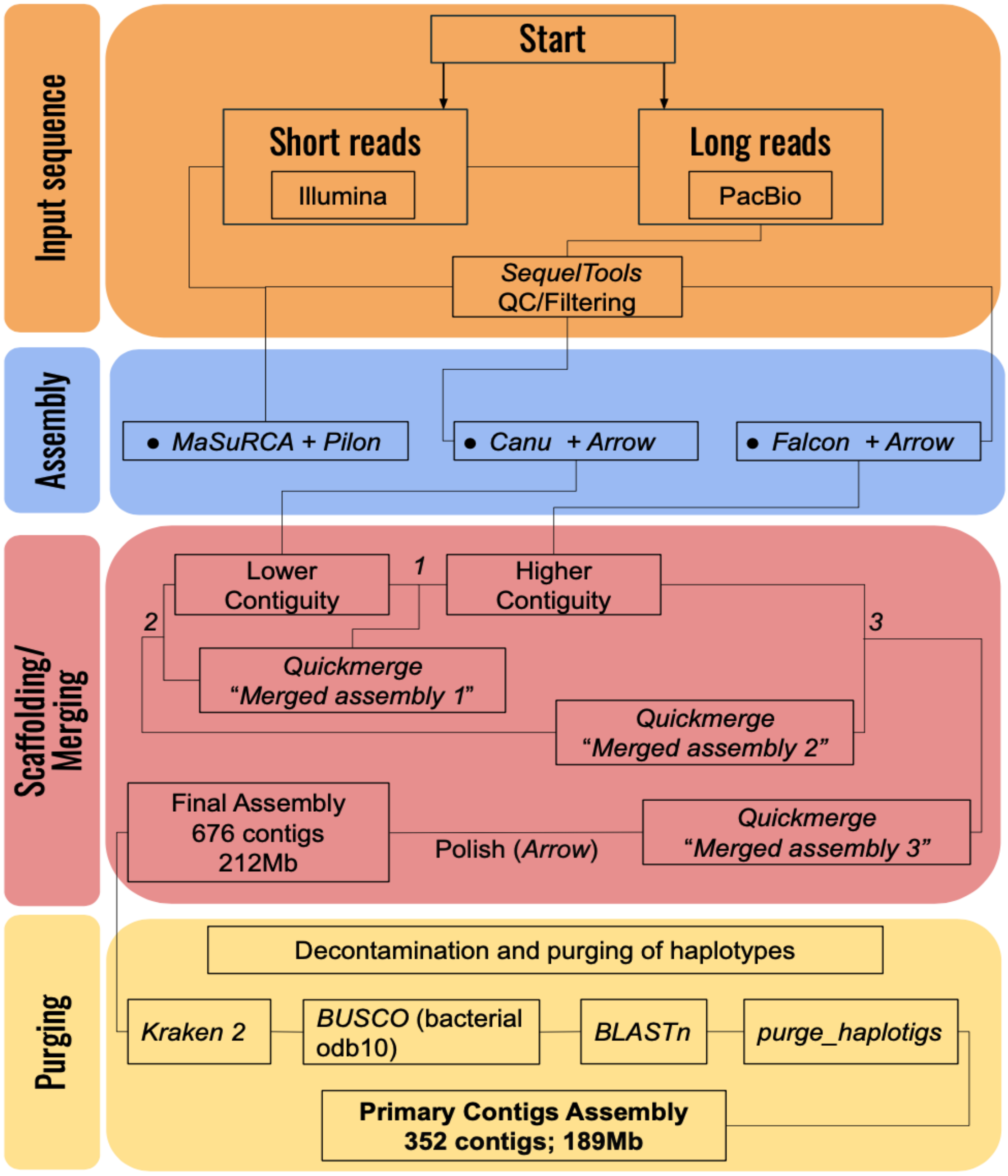

Figure S7. Detailed *de novo* assembly workflow: The best-merged assembly was acquired from the following rounds of alignment. In the first round, Falcon assembly with the genomic size of 208 MB and N50 of 1.9 MB was taken as a reference, and Canu assembly with the genomic size of 199 MB and N50 of 1.8 MB was taken as a query to obtain the first merged assembly, hereafter named *Merged assembly 1*. To have higher basecall accuracy the second round was performed, where Canu assembly was taken as query and *Merged assembly 1* was taken as reference resulting in the second merged assembly, hereafter named *Merged assembly 2*. The third and final round of merging alignment was performed to further increase assembly contiguity. Where *Merged assembly 2* was taken as a reference and Falcon assembly was taken as a query which resulted in the final merged assembly: *Merged assembly 3*.

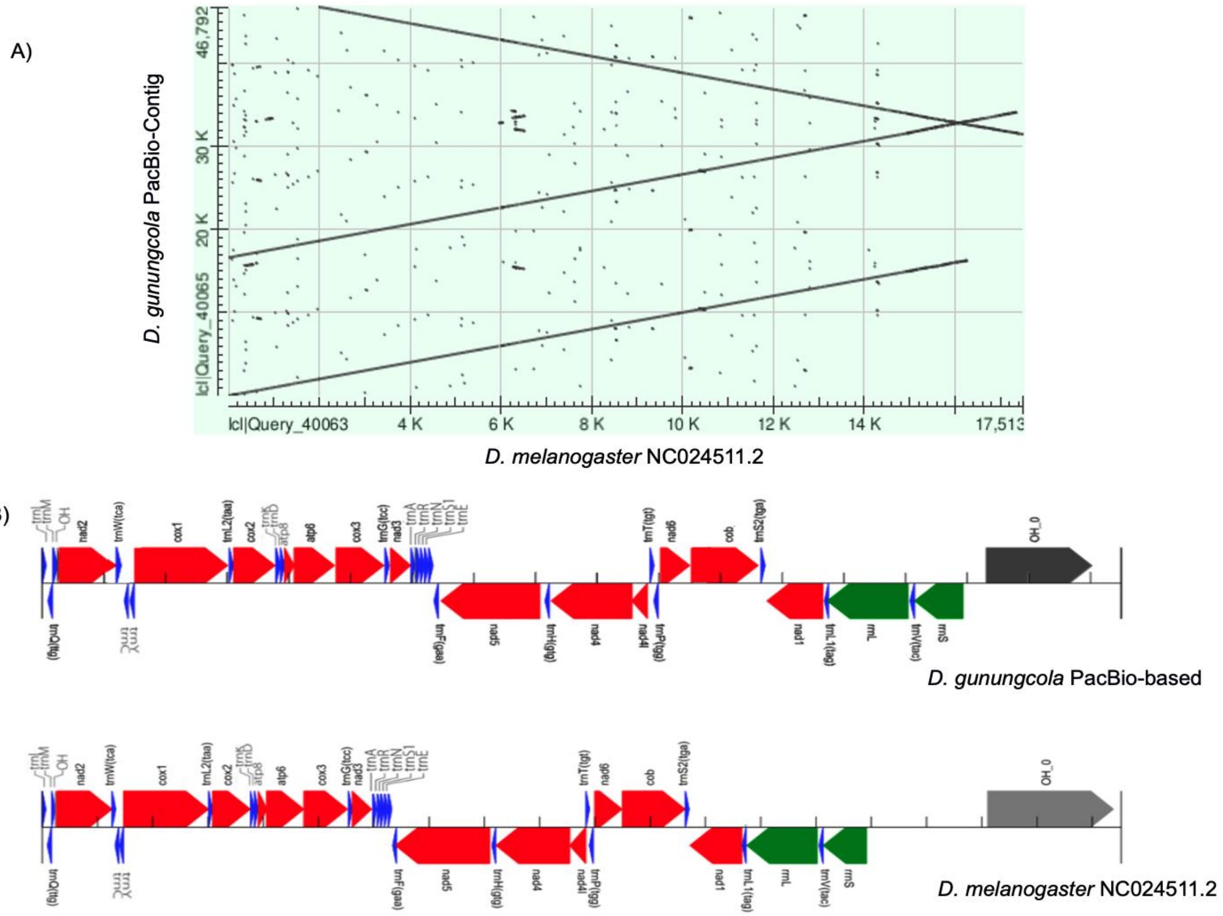

Figure S8. Identification and annotation of *D. gunungcola* mitochondrial genome (JAMKOV010000352 in NCBI database). A) *D. melanogaster* mitochondrial genome aligned to a 46,961-bp long contig of *D.gunungcola* by MegaBLASTn search. B) *D. gunungcola* and *D. melanogaster* mitochondrial genome annotated by MITOS-2. The plot in A) was generated in BLASTu suits and the graph in B) was generated in MITOS-2.

Table S1: Missing and fragmented BUSCO's genes in two assembled *D. gunungcola* genomes (current: GCA_025200985.1 and D_gunungcola_WGS_0.1 GenBank assembly accession: GCA_011057485.1) and *D. melanogaster* r6.36 (Dmel_r6_36). The current *D. gunungcola* assembly recovered 27 BUSCOs that were missing in the previous assembly. But 18 BUSCOs found in the previous assembly are missing in our assembly.

BUSCO ID	Description	*Dgun (this study) (29 Missing) (12 fragmented)*	*D_gun_WGS_0.1 (38 missing) (17 fragmented)*	*Dmel r6.36 (15 missing) (5 fragmented)*
11078at7147	Zinc finger, RING-CH-type	Complete	Complete	Fragmented
12063at7147	Glycerol-3-phosphate dehydrogenase	Complete	Complete	Missing
12741at7147	5'-Nucleotidase, C-terminal	Complete	Complete	Missing
23969at7147	Aromatic amino acid hydroxylase	Complete	Complete	Missing
31450at7147	metallophosphoesterase 1 homolog	Complete	Complete	Missing
432at7147	Immunoglobulin-like domain	Complete	Complete	Missing
44097at7147	interferon-related developmental regulator 2	Complete	Complete	Missing
53285at7147	Uncharacterized protein, isoform A	Complete	Complete	Missing
70512at7147	NHP2-like protein 1 homolog	Complete	Complete	Missing
72609at7147	bis(5'-nucleosyl)-tetraphosphatase	Complete	Complete	Missing
10672at7147	catenin delta-2	Complete	Fragmented	Complete
1388at7147	Nipped-B protein	Complete	Fragmented	Complete
14825at7147	ecotropic viral integration site 5 ortholog isoform X1	Complete	Fragmented	Complete
15520at7147	NADPH--cytochrome P450 reductase	Complete	Fragmented	Complete
18097at7147	alsin homolog	Complete	Fragmented	Complete
28713at7147	WD40/YVTN repeat-like-containing domain superfamily	Complete	Fragmented	Complete
46041at7147	transport and Golgi organization protein 2 isoform X1	Complete	Fragmented	Complete
46546at7147	cytoplasmic phosphatidylinositol transfer protein 1	Complete	Fragmented	Complete
47477at7147	Zinc finger, RING/FYVE/PHD-type	Complete	Fragmented	Complete
48481at7147	Transport and Golgi organization protein 11	Complete	Fragmented	Complete
51566at7147	NAD(P)H-hydrate epimerase	Complete	Fragmented	Complete
7366at7147	catenin alpha	Complete	Fragmented	Complete
76154at7147	U4/U6.U5 small nuclear ribonucleoprotein 27 kDa protein	Complete	Fragmented	Complete
23771at7147	cytochrome P450 315a1, mitochondrial	Complete	Fragmented	Fragmented
43200at7147	protein FAM49B	Complete	Fragmented	Fragmented
11408at7147	Integrator complex subunit 3 homolog	Complete	Missing	Complete
11408at7147	Integrator complex subunit 3 homolog	Complete	Missing	Complete
1636at7147	Dedicator of cytokinesis	Complete	Missing	Complete
18042at7147	Haem peroxidase	Complete	Missing	Complete
18494at7147	Cyclic nucleotide-binding domain	Complete	Missing	Complete
18970at7147	Transcription initiation factor TFIID subunit 5	Complete	Missing	Complete
19314at7147	rho GTPase-activating protein 18	Complete	Missing	Complete
19562at7147	Transferrin	Complete	Missing	Complete
20349at7147	cyclin-dependent kinase 14 isoform X1	Complete	Missing	Complete
24329at7147	Major facilitator superfamily	Complete	Missing	Complete
2483at7147	WD40 repeat	Complete	Missing	Complete
30391at7147	ribose-phosphate pyrophosphokinase 2 isoform X1	Complete	Missing	Complete
33755at7147	26S proteasome regulatory subunit 10B	Complete	Missing	Complete
36427at7147	guanine nucleotide-binding protein subunit alpha homolog	Complete	Missing	Complete
40817at7147	Transketolase, C-terminal domain	Complete	Missing	Complete
41593at7147	WD40-repeat-containing domain	Complete	Missing	Complete
42037at7147	Ribosomal protein S5	Complete	Missing	Complete
42351at7147	Sphingomyelin synthase-related 1	Complete	Missing	Complete
5132at7147	Ankyrin repeat-containing domain	Complete	Missing	Complete
56466at7147	zinc transporter ZIP9-B	Complete	Missing	Complete
56952at7147	mpv17-like protein	Complete	Missing	Complete
5770at7147	Immunoglobulin-like domain	Complete	Missing	Complete

BUSCO ID	Description	*D. gun (this study) (29 Missing) (12 fragmented)*	*D_gun_WGS_0.1 (38 missing) (17 fragmented)*	*D. mela r6.36 (15 missing) (5 fragmented)*
61154at7147	Ribosomal protein S11	Complete	Missing	Complete
63174at7147	EF-hand domain	Complete	Missing	Complete
65216at7147	protein archease-like	Complete	Missing	Complete
12017at7147	Putative pre-mRNA-splicing factor ATP-dependent RNA helicase DHX15	Complete	Missing	Missing
268at7147	TRAF3-interacting protein 1	Complete	Missing	Missing
10531at7147	procollagen-lysine,2-oxoglutarate 5-dioxygenase 1	Fragmented	Complete	Complete
11408at7147	Integrator complex subunit 3 homolog	Fragmented	Complete	Complete
17197at7147	zinc finger CCCH domain-containing protein 13 isoform X1	Fragmented	Complete	Complete
19745at7147	Zinc finger, RING/FYVE/PHD-type	Fragmented	Complete	Complete
20790at7147	Ubiquitinyl hydrolase 1	Fragmented	Complete	Complete
56534at7147	lysosome-associated membrane glycoprotein 5	Fragmented	Complete	Complete
57376at7147	uncharacterized protein LOC106620937	Fragmented	Complete	Complete
66305at7147	coiled-coil domain-containing protein 43	Fragmented	Complete	Complete
72807at7147	B-cell CLL/lymphoma 7 protein family member A	Fragmented	Complete	Complete
34293at7147	cytoplasmic dynein 1 light intermediate chain 1	Fragmented	Complete	Fragmented
36171at7147	acidic fibroblast growth factor intracellular-binding protein	Fragmented	Fragmented	Complete
27639at7147	Sterile alpha motif domain	Fragmented	Fragmented	Fragmented
24614at7147	Chitin binding domain	Missing	Complete	Complete
25096at7147	Major facilitator superfamily	Missing	Complete	Complete
25168at7147	synaptic vesicle glycoprotein 2B	Missing	Complete	Complete
25199at7147	Proton-coupled amino acid transporter 1	Missing	Complete	Complete
40519at7147	protein gustavus isoform X1	Missing	Complete	Complete
51667at7147	Short-chain dehydrogenase/reductase, conserved site	Missing	Complete	Complete
5264at7147	Histone deacetylase	Missing	Complete	Complete
52940at7147	peroxisomal membrane protein PEX14	Missing	Complete	Complete
54524at7147	ribosome maturation protein SBDS	Missing	Complete	Complete
56727at7147	Tetratricopeptide repeat	Missing	Complete	Complete
6029at7147	integrator complex subunit 6	Missing	Complete	Complete
60441at7147	gamma-interferon-inducible lysosomal thiol reductase	Missing	Complete	Complete
61280at7147	alpha-ketoglutarate-dependent dioxygenase alkB homolog 4	Missing	Complete	Complete
61425at7147	ATP synthase, F0 complex, subunit B	Missing	Complete	Complete
63025at7147	Glycine cleavage system H protein	Missing	Complete	Complete
63174at7147	EF-hand domain	Missing	Complete	Complete
8971at7147	Coatomer subunit beta	Missing	Complete	Complete
9202at7147	syntaxin-18	Missing	Complete	Complete
13402at7147	CRAL-TRIO lipid binding domain	Missing	Missing	Complete
13548at7147	Sterile alpha motif domain	Missing	Missing	Complete
22307at7147	protein argonaute-3	Missing	Missing	Complete
39601at7147	carbonic anhydrase 2	Missing	Missing	Complete
40116at7147	putative serine/threonine-protein kinase STE20-like	Missing	Missing	Complete
64099at7147	thioredoxin-like protein 4A	Missing	Missing	Complete
7592at7147	Zinc finger protein	Missing	Missing	Complete
10022at7147	C-terminal PH-like domain	Missing	Missing	Missing
57639at7147	uncharacterized protein LOC108029449	Missing	Missing	Missing
74791at7147	Nuclear RNA-splicing-associated protein, SR-25	Missing	Missing	Missing
75954at7147	Ubiquitin-related modifier 1	Missing	Missing	Missing

Table S2. Statistics of repetitive sequences in *D. gunungcola*.

**Description**	***D. gunungcola***	
# Sequences	352	
Total length:	189859453 bp	
GC%	39.78	
Repeated Element Category	Length occupied (bp)	%
Total bases masked:	54,443,610	28.68
**Retroelements**	**39,571,998**	**20.84**
SINEs:	0	0.00
Penelope	349,455	0.18
LINEs:	9588369	5.05
CRE/SLACS	0	0.00
L2/CR1/Rex	3,250,548	1.71
R1/LOA/Jockey	678,330	0.36
R2/R4/NeSL	30,402	0.02
RTE/Bov-B	40,515	0.02
L1/CIN4	0	0.00
**LTR elements:**	**29,983,629**	**15.79**
BEL/Pao	7,953,327	4.19
y1/Copia	607,412	0.32
Gypsy/DIRS1	21,422,890	11.28
Retroviral	0	0.00
**DNA transposons**	**3,069,593**	**1.27**
hobo-Activator	586,246	0.31
Tc1-IS630-Pogo	625,068	0.33
En-Spm	0	0.00
MuDR-IS905	0	0.00
PiggyBac	46,925	0.02
Tourist/Harbinger	208,022	0.11
Other (Mirage, P-element; Transib)	258,108	0.14
**Rolling-circles transposons**	**5,089,882**	**2.68**
**Unclassified:**	**709,438**	**0.37**
**Small RNA:**	**32,439**	**0.02**
**Satellites:**	**1,118,644**	**0.59**
**Simple repeats:**	**4,106,341**	**2.16**
**Low complexity:**	**745,275**	**0.39**

Table S3. List of OR genes in 9 *Drosophila* species. The number of OR genes is indicated for each species. * denotes there was a wrong annotation of gene model in *D. gunungcola* or *D. elegans* and the number has been corrected after manual inspection. 0 in blue and red denotes pseudogene and complete loss, respectively.

Gene name	*Dmel*	*Dsec*	*Dsim*	*Dere*	*Dyak*	*Dsuz*	*Dgun*	*Dele*	*Dana*
Or1a	1	1	1	1	1	1	1	1	1
Or2a	1	1	1	1	1	1	1	1	1
Or7a	1	1	1	1	1	1	1	1	1
Or9a	1	1	1	1	1	1	1	1	1
Or10a	1	1	1	1	1	1	1	1	1
Or13a	1	0	1	1	1	1	1	1	1
Or19a/b	2	1	1	1	1	2	1	1	1
Or22a/b	2	1	2	2	1	2	2*	2	4
Or22c	1	1	1	1	1	1	1	1	1
Or23a	1	1	1	1	1	2	1	1	1
Or30a	1	1	1	1	1	1	1	1	1
Or33a	1	1	1	1	1	1	2*	2*	2
Or33b	1	1	1	1	1	1	1	1	3
Or33c	1	1	1	1	1	1	**0**	1	1
Or35a	1	1	1	1	1	1	1	1	1
Or42a	1	1	1	1	1	1	1	1	1
Or42b	1	1	1	1	1	1	1	1	1
Or43a	1	1	1	1	1	1	1	1	1
Or43b	1	1	1	1	1	1	1	1	1
Or45a	1	1	1	1	1	1	1	1	1
Or45b	1	1	1	1	1	1	1	1	1
Or46a	1	1	1	1	1	1	1	1	1
Or47a	1	1	1	1	1	1	1	1	1
Or47b	1	1	1	1	1	1	1	1	1
Or49a	1	1	1	1	1	2	**0**	**0**	1
Or49b	1	1	1	1	1	1	1	1	1
Or56a	1	1	1	1	1	1	1	1	1

**Gene name**	***Dmel***	***Dsec***	***Dsim***	***Dere***	***Dyak***	***Dsuz***	***Dgun***	***Dele***	***Dana***
Or59a	1	1	1	1	1	2	1	1	1
Or59b	1	1	1	1	1	1	1	1	1
Or59c	1	1	1	1	1	2	1	1	1
Or63a	1	1	1	1	1	1	1	1	1
Or65a	1	1	1	1	1	1	1	1	3
Or65b/c	2	1	2	2	3	1	2*	2*	0
Or67a	1	1	1	1	2	5	**0**	**0**	1
Or67b	1	1	1	1	1	1	1	1	1
Or67c	1	1	1	1	1	1	1	1	1
Or67d	1	1	1	1	1	1	1	1	1
Or69a	1	1	1	1	1	1	1	1	1
Or74a	1	1	1	1	1	0	1	1	1
Or82a	1	1	1	1	1	1	1	1	1
Or83a	1	1	1	1	1	1	1	1	1
Or83c	1	1	1	1	1	1	1	1	1
Or85a	1	1	1	1	1	1	1	1	0
Or85b/c	2	2	2	2	2	2	2	2	2
Or85d	1	1	1	1	1	1	1	1	1
Or85e	1	1	1	1	1	1	1	1	1
Or85f	1	1	1	1	1	1	1	1	1
Or88a	1	1	1	1	1	1	1	1	1
Or92a	1	1	1	1	1	1	1	1	1
Or94a	1	1	1	1	1	1	1	1	1
Or94b	1	1	1	1	1	1	1*	1	1
Or98a	1	1	1	1	2	1	1	1	3
Or98b	1	0	1	1	1	1	1	1	1
ORCO	1	2	1	1	1	2	1	1	1
**Total number**	**58**	**54**	**57**	**57**	**59**	**65**	**55**	**56**	**63**

Table S4. List of IR genes in 9 *Drosophila* species. The number of IR genes is indicated for each species. * denotes there was a wrong annotation of gene model in *D. gunungcola* or *D. elegans* and the number has been corrected after manual inspection. 0 in blue and red denotes pseudogene and complete loss, respectively.

Gene name	*Dmel*	*Dsec*	*Dsim*	*Dere*	*Dyak*	*Dsuz*	*Dgun*	*Dele*	*Dana*
Ir7a	1	1	1	1	1	1	1	1	1
Ir7b	1	1	1	1	1	1	1	1	1
Ir7c	1	1	1	1	1	1	1	1	1
Ir7d	1	1	1	1	1	1	1	1	1
Ir7e	1	1	1	1	1	1	1*	1	1
Ir7f	1	1	1	1	1	1	**0**	1	1
Ir7g	1	1	1	1	1	1	1*	1	1
Ir8a	1	1	1	1	1	1	1	1	1
Ir10a	1	1	1	1	1	1	1	1	1
Ir11a	1	1	1	1	1	1	**0**	1	1
Ir20a	1	1	1	1	1	1	1	1	0
Ir21a	1	1	1	1	1	1	1	1	1
Ir25a	1	1	1	1	1	1	1	1	1
Ir31a	1	1	1	1	1	1	1	1	1
Ir40a	1	1	1	1	1	1	1	1	1
Ir41a	1	1	1	1	1	1	1	1	1
Ir47a	1	0	1	1	1	1	**0**	1	1
Ir48a/b/c	2	2	3	2	3	2	2	2	2
Ir51a	0	1	1	1	1	1	1	1	1
Ir51b	1	1	1	1	1	0	1	1	1
Ir52a/b/c/d/e/f	4	4	6	5	5	5	5*	5*	4
Ir54a	1	1	1	1	1	1	1	1	1
Ir56a	1	1	1	1	1	1	1	1	1
Ir56b/c/d	3	3	3	3	3	3	3	3	2
Ir56e	0	0	0	0	0	0	1	1	1

Gene name	*Dmel*	*Dsec*	*Dsim*	*Dere*	*Dyak*	*Dsuz*	*Dgun*	*Dele*	*Dana*
Ir60a	1	1	1	1	1	1	1	1	1
Ir60b/c/d/f	2	2	3	4	3	3	1*	1*	4
Ir60e	1	1	1	1	1	1	2*	2*	1
Ir62a	1	1	1	1	1	1	1	1	1
Ir64a	1	1	1	1	1	1	1	1	1
Ir67a/b/c	3	2	3	3	3	3	3	3	2
Ir68a	1	1	1	1	1	1	1	1	1
Ir68b	1	1	1	1	1	1	1	1	1
Ir75a/b/c/d	4	3	4	4	4	4	4	4	4
Ir76a	1	1	1	1	1	1	1	1	1
Ir76b	1	1	1	1	1	1	1	1	1
Ir84a	1	1	1	1	1	1	1	1	1
Ir85a	1	1	1	1	1	1	1	1	1
Ir87a	1	1	1	1	1	1	1	1	1
Ir92a	1	1	1	1	1	1	1	1	1
Ir93a	1	1	1	1	1	1	1	1	1
Ir94a/b/c	3	1	3	2	3	1	**0**	**0**	3
Ir94d/e	2	2	2	2	2	2	**0**	2*	2
Ir94f/g/h	3	3	3	3	3	3	3	3	3
Ir100a	1	1	1	1	1	1	1	1	1
**Total Number**	**60**	**56**	**65**	**63**	**64**	**60**	**55**	**60**	**61**

Table S5. Data statistics of RNA-seq dataset from *D. gunungcola*. The assembled mRNA library was used for genome annotation.

Samples	Raw reads	Raw reads Yield (GB)	Filtered reads (by FastP)	% of filtered reads with Q30	Assembled mRNA library: *Trinity* (MB)
Adult male	88,556,882	26.28	88,208,388	95.24	123.9
Adult female	163,879,654	48.58	163,356,466	95.41	117.7
Mixed-instar larva	84,518,508	24.68	84,241,504	95.78	95
Male pupa	92,627,064	26.89	92,246,506	95.12	142.2
Female pupa	86,289,804	22.94	85,997,762	95.61	133.3

Table S6. Data statistics of raw PacBio reads from three SMRT Cells.

Description	SMRT Cell 1	SMRT Cell 2	SMRT Cell 3
Number of CLR	85,868	593,928	561,879
Total CLR bases	766,949,467	5,375,978,518	5,289,664,859
Mean Read length of CLR	8,945	9,055	9,418
N50 CLR length	23,505	18,827	17,015
Number of filtered subreads	104,551	633,367	5,99,656
Total filtered subread bases	606,119,588	5,119,698,166	5,034,716,844
Mean Read length of filtered subreads	7,336	8,488	8,821
N50 filtered subread length	16,366	11,955	12,078

Table S7. Assessment of assemblies acquired by five genome assemblers.

Assembler	Assembly type	Assembly size (Mb)	#contigs	N50 (Mb)	Longest contig (Mb)	GC content (%)	Assembly completeness (BUSCOs%)	#N's per 100 Kbp
Canu	PacBio -only	199.5	834	1,646,760	28,294,737	39.79%	98.4	0.00
Falcon	PacBio- only	213.1	673	1,939,263	29,368,726	39.70%	98.6	0.00
HGAP-4	PacBio- only	206.13	780	1,122,981	27,427,377	39.70%	97.8	0.00
MaSuRCA	Hybrid	208.09	3,146	399,971	3,245,546	39.55%	94.7	316.51
Quickmegre	Merged (Canu+Falcon)	212.09	676	2,135,404	31,037,916	39.78%	98.9	0.00

Table S8. An overview of *D. gunungcola* transcript annotation rounds

Description	Round 1	Round 2	**Round 3**	Round 4	Round 5
Annotated transcript completeness (BUSCO n=3285)	2904	2729	**2901**	2734	2729
Complete and single-copy	2813	2650	**2810**	2650	2646
Complete and duplicated	91	79	**91**	84	83
Fragmented	77	99	**90**	93	94
Missing	304	457	**294**	458	462
Annotated protein completeness (BUSCO n=3285)	2878	2801	**2884**	2801	2794
Complete and single-copy	2860	2781	**2869**	2783	2776
Complete and duplicated	18	20	**15**	18	18
Fragmented	154	117	**157**	122	125
Missing	243	367	**7244**	362	368
Predicted # of gene	16,558	15,063	**14,265**	14,327	14,023
Predicted # of CDSs	17,543	16,002	**14,703**	14,798	14,602
Mean gene length (bp)	3,583.22	4,187.91	**4,248.19**	4,203.47	4,201.09
AED Mean	0.34	0.15	**0.36**	0.41	0.4
AED Median	0.2	0.20	**0.12**	0.13	0.18
Gene models with AED of ≤ 0.5 (%)	99.3	91.0	**96.8**	94	94
